# Supplementary material for: Qualitative study of healthcare providers’ current practice patterns and barriers to successful rehydration for pediatric diarrheal illnesses in Kenya
Source: PeerJ. 2017 Oct 6;5:e3829. doi: 10.7717/peerj.3829 (PMC5633020; doi:10.7717/peerj.3829)
Supplement: Supplemental Information 2 [file peerj-05-3829-s002.docx]

**Interview #1 – MO at Referral Sick Child**

(Interviewer): You have gone through a scenario about resuscitating a child with dehydration. When you think about treating dehydration, what are the first methods that come to mind? What options do you consider when there are difficulties?

(MO): The first thing is that I should access the IV right away; and then if the child is in shock then you can start with the bolus. If you fail to get the IV right then, you can put the NG tube and then in 20 mls as a bolus as well.

(Interviewer): Have you tried other things as well? Are there other things you would do besides an NG tube?

(MO): Sometimes we can use intraosseus. Sometimes even the cut down.

(Interviewer): What challenges do you face when resuscitating a child with dehydration?

(MO): When they come with the collapsed veins.

(Interviewer): So the collapsed veins are the most difficult thing.

(MO): Yes, they are the most difficult.

(Interviewer): What improves your ability to provide rehydration for children? Are there things that make it better? (MO): Availability of the items you need at the site of resuscitation

(Interviewer): What sort of items

(MO): For example the NG tube, like that time we couldn’t get the right size

For that patient, sometimes you become stranded you have to wait for them

To look for those items

(Interviewer): so at times you don’t have the right size of NG tube

(MO): Yes.

(Interviewer): Tell me about circumstances in which you would use a nasogastric tube for rehydration therapy.

(M0): When you can’t access the IV Line.

(Interviewer): What would make it difficult to use an NG tube?

(MO): Sometimes insertion

(Interviewer): Insertion is sometimes difficult. What would make this process easier?

(MO): When you have the KY jelly, it can move quickly.

(Interviewer): Do you sometimes not have lubrication?

(MO): At that time when I needed it, it was there

(Interviewer): Are there circumstances when you would not use a nasogastric tube for rehydration therapy?

(MO): Yes, incase when you get the intravenous you don’t have to use an NG.

(Interviewer): Are NG tubes available in the setting in which you work?

(MO): They are available.

(Interviewer): Easily available or sometimes?

(MO): It depends with the sizes. They are available depending with the sizes. Sometimes you can’t get the right size when you need it, but they are there

(IF not): Have you ever had NG tubes available there?

(Interviewer): What training or experience have you had with using nasogastric tubes?

Have you used them a lot or a little.

(MO): I have used then a lot in surgical ward with intestinal obstruction

(Interviewer): How familiar are you with the World Health Organization Guidelines for rehydration therapy for diarrheal illnesses?

(MO): I think I still have to go over them again.

(Interviewer): How much training have you had regarding these guidelines?

(MO): Just the general training in the college.

(Interviewer): just general training in the college…….. Okay. Have there been any courses since you started working that go over treatment of diarrhea or resuscitation.

(MO): I am very new in this place. Initially I was in obgy there is cs and whatever, so here I am very new, this is my 4^th^ week so I haven’t gone for any so far to do with rehydration or diarrhea and vomiting.

(Interviewer): How do you think a child’s family would react if you wanted to use an NG tube for rehydrating their child?

(MO): I think I should fast of all inform them and get consent from them and why I am doing it I shouldn’t just go and start without informing them.

(Interviewer): Do you think families are okay with using an NG tube.

(MO): Of course, if you can tell them that you can’t access the IV, and the only chance for this baby to live is NG tube they will accept because they want the baby to live actually.

(Interviewer): So you don’t think they will find it strange, you are not doing enough. (MO): I don’t think so, provided you have given them information on why I am willing to do this.

(Interviewer): Yer, okay…………very good that’s the end of the survey. Thank you.

(MO): Thank you.

**INTERVIEW #2 CO AT REFERRAL HOSPITAL**

(INTERVIEWER1): You have gone through a scenario about resuscitating a child with dehydration. When you think about treating dehydration, what are the first methods that come to mind?

(CO): We usually classify fast to see what grade of dehydration it is where there are around four types there is no dehydration with history of diarrhea and vomiting

Some dehydration, severe dehydration and when severe it gets to shock. So each

Has different management.

(Interviewer2): So for the severe dehydration what would you do? Like what are the

Things that you would try, you certainly mentioned IV.

(CO): Yer, severe warrants for IV therapy and we always use zinc

Sulphate. It’s a recommendation, it reduces the frequency of

Diarrhea, also investigate and treat the cause.

(Interviewer1): What options do you consider when there are difficulties?

(CO): For example?

(Interviewer1): When you are not able to get the line or any other difficulty.

(CO): Like we have said, we usually call one another, and attempt if it fails

………. At times there is an option of NG tube but the challenge

Is there is vomiting. The last option is intraosses, which is a remedy

As we continue looking for the IV

(Interviewer): What challenges do you face when resuscitating a child with dehydration?

(CO): Sometimes the relatives might not be cooperative, some

Parents don’t let their children to be pricked more than

Once if the line is not obtainable, the IV line is not available

Easily. Another one is ongoing losses, if there is a

Super infection. And if you don’t calculate properly the

Dosage of the amount of fluid to give you might cause Overload.

(Interviewer1): What improves your ability to provide rehydration for children?

(CO): There should be availability for the required options for

Rehydration, like ORS, IV fluids and equipment’s like brannulas

(Interviewer2): are there times you don’t have availability of supplies?

(CO): of late no, there usually there. We don’t have problems as Per now.

(Interviewer): In the case scenario, you did not suggest using a nasogastric tube for rehydration therapy. What were your reasons for this?

(CO): For suggesting nasogastric?

(Interviewer1): For not suggesting in the fast place.

(CO): At some point I mentioned that it is an option, although it can

Challenging if vomiting is continuous.

(Interviewer1): in the fast place when I was asking you questions you didn’t

Suggest it, so what was your reason for not suggesting it At that time?

(CO): From the scenario there is history of diarrhea and vomiting,

So I considered with that vomiting it might not help us that much

Though it’s a remedy it can help as you continue accessing The IV line.

(Interviewer1): Are there circumstances where you wouldn’t use nasogastric Tube for rehydration?

(CO): Like contraindications or something?

(Interviewer): Yer

(CO): I don’t think so.

(Interviewer): Like in this case where you said vomiting

(CO): Unless the patient is in shock you give an ng tube….

Where there is altered consciousness they

Can vomit and aspirate.

(Interviewer): Are NG tubes available in the setting in which you work?

(CO): Yes.

(Interviewer1): What would it make it difficult to use an NG tube?

(CO): The state of consciousness of the patient, although

Conscious patients might not be cooperative the procedure

Is uncomfortable and sometimes it can go a wrong way to the

Bronchi of which it needs one to be very keen so that you

Don’t. There are ways you can test to see whether you are

In the right place.

(Interviewer1): What would make the process easier?

(CO): Training.

(Interviewer1): What training or experience have you had with using nasogastric tubes?

(CO): Am a trained pediatrician and I got a lot of experience

In Kenyatta national hospital where we used so much

So much of it.

(Interviewer1): So experience and in college.

(Interviewer2): Have you used NG tubes many times?

(CO): Yer, especially for the surgical cases we commonly use it

Where there is intestinal obstruction.

(Interviewer1): How familiar are you with the World Health Organization Guidelines for rehydration therapy for diarrheal illnesses?

(CO): Am familiar.

(Interviewer1): How much training have you had regarding these guidelines?

(CO): It’s difficult to measure but we covered during higher diploma training

It was a topic; we also did in etat emergency triage, which it covers.

(Interviewer1): How do you think a child’s family would react if you wanted to use an NG tube for rehydrating their child?

(CO): Most of them get worried, the way at times they get worried when you fix an oxygen cannula when you put it they think it’s so severe most of them are actually not comfortable, they dread it’s a severe state others can even cry, though

They don’t refuse, they become desperate.

(Interviewer2): How do they take it when you explain it to them?

(CO): They take it positively, they don’t refuse.

(CO): So if they understand why you are doing it then they are okay

(Interviewer): So that’s it, thank you.

**INTERVIEW#3 CLINICAL OFFICER AT A REFARAL HOSPITAL.**

(Interviewer): You have gone through a scenario about resuscitating a child with dehydration. When you think about treating dehydration, what are the first methods that come to mind? What options do you consider when there are difficulties?

(CO): Giving the per oral then giving the intravenous fluid.

(Interviewer): What options do you consider when there are difficulties?

(CO): If the child is able to take orally, if there is vomiting……… I will also

Consider the severity of the dehydration if there is some dehydration,

No dehydration or severe.

(Interviewer): So when rehydrating the child what options would you consider?

(CO): Difficulties in term of?

(Interviewer): while rehydrating the child, any difficulties you encounter.

(CO): For example, if there is difficulty of ………. The child can’t retain then I will

Go for IV fluids, if the difficulty is in getting an IV line then I will go for intaosseous.

(Interviewer): What challenges do you face when resuscitating a child with dehydration?

(CO): Maybe when the child cannot retain orally, another challenge is when they cannot afford to acquire the IV fluids or they cannot afford to get the per oral

Rehydrations.

(Interviewer): Okay so the cost. Like in our case scenario we had a child who was

Severely dehydrated what challenges would you face would you face when resuscitating this child?

(C0): Getting an IV line maybe a problem because the child is severely dehydrated

Now that the child is severely dehydrated the only option I will have with this child is Intravenous, the child will not cope with oral rehydration.

(Interviewer): What improves your ability to provide rehydration for children? (CO): The severely dehydrated child will receive IV fluids while the dehydrated child will be rehydrated orally.

(Interviewer): In the case scenario, you did not suggest using a nasogastric tube for rehydration therapy. What were your reasons for this?

(CO): Nasogastric can be used, but it depends if the child is vomiting

(Interviewer): Tell me about circumstances in which you would use a nasogastric tube for rehydration therapy. What would make it difficult to use an NG tube? What would make this process easier?

(CO): Circumstances where I would use a nasogastric tube are when a child is dehydrated able to take orally but is not vomiting. Difficulty in use would be when a child is fully conscious. The process would be easier if the child is not vomiting.

(Interviewer): Are there circumstances when you would not use a nasogastric tube for rehydration therapy?

(CO): When the child is severely vomiting.

(Interviewer): Are NG tubes available in the setting in which you work? (IF not): Have you ever had NG tubes available there?

(CO): Yes they are available.

(Interviewer): What training or experience have you had with using nasogastric tubes?

(CO): I have been using NGTs during my training and work experience.

(Interviewer): How familiar are you with the World Health Organization Guidelines for rehydration therapy for diarrheal illnesses? How much training have you had regarding these guidelines?

(CO): I have no training in these guidelines.

(Interviewer): How do you think a child’s family would react if you wanted to use an NG tube for rehydrating their child?

(CO): Most parents do not accept NGT as a priority in rehydrating.

What are the biggest barriers to rehydrating children – education? Procedural skills? (Do you know how to put in an NG? What size for what kid?) Availability of NG tube sizes? Cultural beliefs? Do you think NGs are as good as IVs?

**INTERVIEW#4. NURSE IN A REFFARAL HOSPITAL.**

(Interviewer):You have gone through a scenario about resuscitating a child with dehydration. When you think about treating dehydration, what are the first methods that come to mind?

(Nurse): The first methods that come to mind are ORS and intravenous fluids.

(Interviewer): What options do you consider when there are difficulties?

(Nurse): In dehydration when you are not able to get a line or the intraosseous

You can fix an NG tube and give ORS

(Interviewer): What challenges do you face when resuscitating a child with dehydration? (Nurse): Like this intraosseous lines I think most of us are not able to fix so we have to get somebody from somewhere to come and do it which leads to time wasting.

(Interviewer1): Which people have the training to do intraosseous?

(Nurse): Here there was an MO who used do it but currently she is not here she went to school and moved to another department, the rest have not been trained to fix an ion.

(Interviewer): What improves your ability to provide rehydration for children?

(Nurse): Probably training.

(Interviewer): In the case scenario, you did not suggest using a nasogastric tube for rehydration therapy. What were your reasons for this?

(Nurse): I think I just forgot, even before you try intraosseous you can put an NG tube and give ORS.

(Interviewer): Tell me about circumstances in which you would use a nasogastric tube for rehydration therapy.

(Nurse): If you fail to get an IV access you can use an NG tube.

(Interviewer): What would make it difficult to use an NG tube?

(Nurse): I don’t think there is anything difficult.

(Interviewer): What would make this process easier?

(Nurse): I think the process is just easy, so there is no hindrance.

(Interviewer): Are there circumstances when you would not use a nasogastric tube for rehydration therapy?

(Nurse): Yes, like an unconscious patient.

(Interviewer): Are NG tubes available in the setting in which you work? (IF not): Have you ever had NG tubes available there?

(Nurse): Yer they are available.

(Interviewer): All the tome or sometimes?

(Nurse): All the time, though of late we have been getting the right sizes there was a time we couldn’t the small ones.

(Interviewer1): Do you think most people know what sizes to put in children, or is it a problem.

(Nurse): I think to some people they are not sure of which size as per age, because you find people asking one another, so they have no confidence that this is the right one for that particular age.

(Interviewer): What training or experience have you had with using nasogastric tubes?

(Nurse): There is no training only what I learned while I was in college.

(Interviewer): And the experience you have had while working.

(Nurse): I have fixed several of them and I have not encountered any difficulties fixing an NG tube.

(Interviewer): How familiar are you with the World Health Organization Guidelines for rehydration therapy for diarrheal illnesses?

(Nurse): We have not been trained on WHO guidelines.

(Interviewer): So how would you rate your familiarity with the WHO guidelines?

(Nurse): I would say minimal.

(Interviewer): So you have said you don’t have any training.

(Nurse): Yes.

(Interviewer): How do you think a child’s family would react if you wanted to use an NG tube for rehydrating their child?

(Nurse): Some of them who have never experienced their child being fixed an NG tube are reluctant okay they look like they are scared but with reassurance then they don’t mind they take it positively, but those who have gone through the experience before are comfortable with it.

(Interviewer1): What do you think would make the resuscitation better?

(Nurse): Like here?

(Interviewer1): Yer, so that children get their fluids right away.

(Nurse): I think the best thing is training the staff.

(Interviewer): Procedural skills or do you think its education or availability or there are other things that people just believe or cultural expectations?

(Nurse): The staffs?

(Interviewer1): Yer.

(Nurse): I think training would assist, and maybe we should just make it the routine to follow the procedure when we get such cases, then we just get used to. Maybe we put some emphasis if its fixing an NG tube let’s say we should always if you fail maybe to get the line why not fix the NG tube and we try to do it practically then we get used to.

What are the biggest barriers to rehydrating children – education? Procedural skills? (Do you know how to put in an NG? What size for what kid?) Availability of NG tube sizes?

**INTERVIEW# 5 Clinical officers at a referral hospital.**

(Interviewer): You have gone through a scenario about resuscitating a child with dehydration. When you think about treating dehydration, what are the first methods that come to mind?

(CO): Oral rehydration and if not retaining you go ahead and give IV rehydration. And encourage a lot of breast feeding.

(Interviewer): What options do you consider when there are difficulties?

(CO): Like in oral rehydration if the child is not retaining and cannot retain ORS I’d rather go to IV rehydration, to reverse the dehydration then if IV rehydration is difficult like I don’t have fluids or I don’t have the giving sets necessary for IV rehydration then I’ll refer.

(Interviewer): So the difficulties sometimes can be availability of resources.

What challenges do you face when resuscitating a child with dehydration?

(CO): The attitude the mother has, we have encountered mothers or parents who have issues with IV rehydration they associate dehydration or diarrhea and vomiting with witchcraft so that they have the belief that when you give the baby IV fluids the child will die, so maybe I think that is one of the issues. Another issue is parents coming when the child is severely dehydrated and accessing a vein is difficult, so that you really take a lot of time, and this time the baby deteriorates, another one is shortage of staff so that at times we have a bulky queue outside here and a child with dehydration come. You give priority on that baby and you end up spending so much time on that baby and so the queue really waits and client satisfaction becomes an issue.

(Interviewer): So there are issues with cultural beliefs and time of the arrival of the patient.

What improves your ability to provide rehydration for children?

(CO): Early presentation to the hospital, team work at the facility so that when we identify dehydration everyone tries to play a role.

(Interviewer): In the case scenario, you did not suggest using a nasogastric tube for rehydration therapy. What were your reasons for this?

**OR** if participant choose an NG tube in the scenario: What were your reasons for choosing an NG tube in the case scenario for rehydration therapy?

(CO): Well we usually use a nasogastric tube when we see the baby is worsely off and we are not getting anywhere, and we see no help soon enough.

(Interviewer): So you use nasogastric tubes as a last option?

(CO): Not as a last option but we use it as an alternative method for IV rehydration only if the IV rehydration is not accessible but we rarely use it.

(Interviewer): Tell me about circumstances in which you would use a nasogastric tube for rehydration therapy.

(CO): This are circumstances when a child is not able to take orally and you are not able to access an IV line and the baby is severely dehydrated.

(Interviewer): What would make it difficult to use an NG tube?

(CO): At times getting the right size of the tube has become a major challenge and another thing is you find at times this child is on oxygen and you find it difficult to give an NG tube via the other nostril but I don’t think that will warrant a reason enough not to use a nasogastric tube,

(Interviewer): What would make this process easier?

(CO): Making the ORS available and ready.

(Interview): Are there circumstances when you would not use a nasogastric tube for rehydration therapy?

(CO): When the baby is able to take orally and can retain ORS. Also in severe dehydration where you think oral dehydration will take long to reverse the dehydration.

(Interviewer): Are NG tubes available in the setting in which you work?

(CO): Yes we have NG tubes but at times we don’t have the right sizes

(Interview): What training or experience have you had with using nasogastric tubes?

(CO): Experiences when I have used an NG tube is where we have IO and occasionally when we have shock and accessing an IV line has been difficult

(Interviewer): How familiar are you with the World Health Organization Guidelines for rehydration therapy for diarrheal illnesses?

(CO): Not so vast but I guess I know the basics.

(Interviewer): How much training have you had regarding these guidelines?

(CO): None.

(Interviewer): How do you think a child’s family would react if you wanted to use an NG tube for rehydrating their child?

(CO): They get scared thinking there baby is very sick and at times they may not cooperate but on explanation and when you engage them you tell them you are only using that meanwhile as you access the IV line they may take it well. (Interviewer): So what are the biggest barriers to dehydration do you face in this setting.

(CO): We don’t have enough rooms and late presentation of the baby and sometimes lack of cooperation from the other staff and I would attribute that to staff shortage

(Interviewer1): What do you think people think about using NGs?

(CO): People don’t take it well because they think by the time the baby has pipes in their nostrils that baby is very sick and is dying the next minute so they would fear coming to the hospital if they think my baby will have tubes in the nostril. (Interviewer1): Do you think providers think NGs are as good as IV?

(CO): We don’t think they are good as IVs because we have this opinion they are slow it has to go through the GI and be absorbed.

(Interviewer): Do you think the perception of NGs is very poor because they are used very late.

(CO): Probably, We always resort to the NGs as the last resort.

**INTERVIEW #6 NURSE IN A REFERRAL HOSPITAL**

(Interviewer): You have gone through a scenario about resuscitating a child with dehydration. When you think about treating dehydration, what are the first methods that come to mind?

(Nurse): ORS and then IV.

(Interviewer): What options do you consider when there are difficulties?

(Nurse): I think it’s just that one for intraosseous .

(Interviewer): What challenges do you face when resuscitating a child with dehydration? (Nurse): That one of the failure of not getting the IV and sometimes they come when they are severely dehydrated and they die

(Interviewer):What improves your ability to provide rehydration for children?

(Nurse): Sometimes we run short of the equipment so I think if the hospital is well equipped with the equipment’s we use, and then trainings likes CMEs mostly on dehydration will also help us .

(Interviewer): In the case scenario, you did not suggest using a nasogastric tube for rehydration therapy. What were your reasons for this?

(Nurse): I didn’t remember otherwise it’s also a good option.

(Interviewer): What would make it difficult to use an NG tube?

(Nurse): If the patient is in comma and the position of which the patient cannot the NGT cannot go direct to the abdomen maybe that’s where the problem will come in. (Interviewer): What would make this process easier?

(Nurse): Sometimes there are patients who are unwilling , if they are not unconscious there are those who are unwilling .

(Interviewer): So educating the patient and informing will make it easier, do you think everybody knows how to put NG tubes and the right sizes in which they should use? (Nurse): Yes, that’s why I was saying those trainings and CMEs will help, because not all of us……………….

(Interviewer): Are there circumstances when you would not use a nasogastric tube for rehydration therapy?

(Nurse): If the patient can take it orally or they have tried the IV method and it is working then there is no need for it.

(Interviewer): Are NG tubes available in the setting in which you work?

(Nurse): Yer they are available.

(Interviewer): So they are always available?

(Nurse): Sometimes we miss them, sometimes we miss the right sizes.

(Interviewer): So they are available depending on the sizes.

(Nurse): Yes.

(Interviewer): What training or experience have you had with using nasogastric tubes? (Nurse): I have not gone for any training.

(Interviewer): Experience?

(Nurse): Just from my working place here.

(Interviewer): How familiar are you with the World Health Organization Guidelines for rehydration therapy for diarrheal illnesses?

(Nurse): How do you want me to put it do I explain or do I just say I am familiar. (Interviewer):If you are familiar with its okay. How much training have you had regarding these guidelines?

(Nurse): I have not gone for any trainings but maybe CMEs .

(Interviewer): How do you think a child’s family would react if you wanted to use an NG tube for rehydrating their child?

(Nurse): It depends, some who know about it will take it easy. But others who don’t know they might refuse.

(Interviewer): So if you take consent from them and explain it to them they’ll still refuse.

(Nurse): They will not refuse concerning the state of the patient. If the patient is very sick then they don’t have any option.

(Interviewer): So what are your biggest barriers you face while rehydrating children?

(Nurse): Failure to get the IV lines and sometimes when you miss the brannulas, when things are not available.

(Interviewer): You also mentioned people should be trained. When people come and you want to put NGs on their children are there any cultural beliefs that hinder them? (Nurse): I have not heard or any.

(Interviewer): So do you think NGs are good as IVs?

(Nurse): IVs are better than NGs, IVs go direct while the NGTs go the stomach for absorption, for the IV it works faster.

**INTERVIEW #7 CLINICAL OFFICER AT A REFARAL HOSPITAL**

(Interviewer): You have gone through a scenario about resuscitating a child with dehydration. When you think about treating dehydration, what are the first methods that come to mind?

(CO): IV fluids.

(Interviewer): What options do you consider when there are difficulties?

(CO): ORS through an NGT and then you check the sugars.

(Interviewer): What challenges do you face when resuscitating a child with dehydration?

(C0): One of it will be getting an IV access and the parents are not cooperative, while holding the child.

(Interviewer): So why do you think the parents are not cooperative?

(CO): Maybe they fear their child is dying, some fear seeing their children being pricked

(Interviewer): What improves your ability to provide rehydration for children? (CO): Having the utilities available, and then requiring the cooperation of the parent or the guardian.

(Interviewer): In the case scenario, you suggested using a nasogastric tube after the IV rehydration failed what was your reason for this?

(CO): Because this child is not retaining and since the NG tube is up to the level of the abdomen I don’t think the child will vomit.

(Interviewer): Tell me about circumstances in which you would use a nasogastric tube for rehydration therapy.

(CO): One when you can’t get the IV line and two when you don’t have the fluids available like the ringers lactate so you opt to use the ORS.

(Interviewer): What would make it difficult to use an NG tube?

(CO): Nothing.

(Interviewer): What would make this process easier?

(CO): There is no difficulty when there is an NG tube you just fix it there is no problem.

(Interviewer): Are there circumstances when you would not use a nasogastric tube for rehydration therapy?

(CO): Year when you get an access of an IV line then there is no need of using it.

(Interviewer): Are NG tubes available in the setting in which you work?

(CO): Year.

(Interviewer): They are always available?

(CO): Yes

(Interviewer): What training or experience have you had with using nasogastric tubes?

(CO): We don’t use it more often because mostly we are successful in getting the line.

(Interviewer): So have they given you trainings on NG tubes or just the one you got while in college?

(CO): Year from the training in college and from the experience I get from my work.

(Interviewer): How familiar are you with the World Health Organization Guidelines for rehydration therapy for diarrheal illnesses?

(CO): The one I know is plan A to C.

(Interviewer): So you are familiar with it.

(CO): Yes.

(Interviewer): How much training have you had regarding these guidelines?

(CO): Just from the guideline books in have not attended ant training on this.

(Interviewer): How do you think a child’s family would react if you wanted to use an NG tube for rehydrating their child?

(CO): Some positively some don’t agree.

(Interviewer): And when you take permission from them and explain.

(CO): They accept.

(Interviewer): So what are biggest barriers you face while rehydrating the children?

(CO): Maybe failing to get an IV access and maybe the parent is uncooperative. (Interviewer): Do you think NGs are as good as IV?

(CO): I would prefer IV than the NG tube.

(Interviewer): So that’s the end of the survey and interview. Thank you.

**INTERVIEW #8 CLINICAL OFFICER AT A REFARAL HOSPITAL.**

(Interviewer): You have gone through a scenario about resuscitating a child with dehydration. When you think about treating dehydration, what are the first methods that come to mind?

(CO): IV fluids.

(Interviewer): What options do you consider when there are difficulties?

(CO): Give orally.

(Interviewer): What challenges do you face when resuscitating a child with dehydration?

(CO): Mostly the IV access.

(Interviewer): What improves your ability to provide rehydration for children? (Silence)Okay most of the children come when they are severely dehydrated. Instead of the child coming when they are severely dehydrated what do you think they can do?

(CO): We can educate the mother to give fluids at home and coming early.

(Interviewer): In the case scenario, you did not suggest using a nasogastric tube for rehydration therapy. What were your reasons for this?

(CO): Here we mostly use it for feeding, not for rehydration.

(Interviewer): Tell me about circumstances in which you would use a nasogastric tube for rehydration therapy.

(CO): When IV rehydration fails.

(Interviewer): What would make it difficult to use an NG tube?

(CO): Availability of the tubes.

(Interviewer): Are there circumstances when you would not use a nasogastric tube for rehydration therapy?

(CO): Maybe incases of obstruction.

(Interviewer): Are NG tubes available in the setting in which you work?

(CO): At times.

(Interviewer): What training or experience have you had with using nasogastric tubes?

(CO): No training.

(Interviewer): And experience.

(CO): Only my working experience.

(Interviewer): How familiar are you with the World Health Organization Guidelines for rehydration therapy for diarrheal illnesses?

(CO): The classifications.

(Interviewer): So you are familiar with them. How much training have you had regarding these guidelines?

(CO): None.

(Interviewer): How do you think a child’s family would react if you wanted to use an NG tube for rehydrating their child?

(CO): They always feel scared.

(Interviewer): So after taking consent from them and explaining to them why you are using the NG tube will they be comfortable with it.

(CO): Yes.

(Interviewer): Or they don’t have a choice they just have to accept.

(CO): They just have to accept.

(Interviewer): So what are the biggest barriers you face while rehydrating children?

(CO): Mostly IV access.

(Interviewer): Do you think training would improve, training on the use of nasogastric tubes……… just training of the staff.

(CO): Yes it will.

(Interviewer): So in the case of availability of the nasogastric tubes are they always available in the right sizes?

(CO): They are not available sometimes small sometimes big.

(Interviewer): Are there any cultural beliefs that hinder the parents or clinicians from the use of NG tubes.

(CO): No.

(Interviewer): Do you NGs are as good as IV?

(CO): No.

(Interviewer): Okay. So in your opinion what do you think will improve rehydration for the dehydrated children, according to the difficulties you face? (CO): Maybe education of the mothers about dehydration causes and management.

(Interviewer): So education of the society. What else?

(CO): And availability of the NG tubes. And education of the staff.

(Interviewer): So that the end of the survey and interview. Thank you.

**INTERVIEW #9 AT A REFARAL HOSPITAL**

(Interviewer): You have gone through a scenario about resuscitating a child with dehydration. When you think about treating dehydration, what are the first methods that come to mind?

(CO): You classify the dehydration; put an IV line and giving the required fluids. (Interviewer): What options do you consider when there are difficulties?

(CO): You consult maybe the MO; you have the other colleagues try.

(Interviewer): What challenges do you face when resuscitating a child with dehydration?

(CO): Getting the line, sometimes space in this place, we can have three children the space is too little so having time to do those things can be a challenge.

(Interviewer): What improves your ability to provide rehydration for children? (CO): Maybe having an emergency room, where we can have space, the was wards are like an acute room. Because if we combine all the patients it will be difficult to monitor and manage them. And maybe having the other fluids and other supplies available.

(Interviewer): In the case scenario, you suggested using a nasogastric tube for rehydration therapy, so what were your reasons for choosing the NG tubes?

(CO): Maybe the other methods have failed; maybe the line is not there.

(Interviewer): Tell me about circumstances in which you would use a nasogastric tube for rehydration therapy.

(CO): In case we have failed to get an IV line that’s when I can use it. (Interviewer): What would make it difficult to use an NG tube?

(CO): Maybe getting the right size also putting is a procedure, if you don’t have space and those things it can be difficult to access it because the child is so lethargic.

(Interviewer): What would make this process easier?

(CO): If you have enough space and the supplies are there.

(Interviewer): Are there circumstances when you would not use a nasogastric tube for rehydration therapy?

(CO): If a child has congenital malformations maybe in the throat causing obstruction.

(Interviewer): Are NG tubes available in the setting in which you work?

(CO): They are, but sometimes they are not available according to sizes.

(Interviewer): What training or experience have you had with using nasogastric tubes?

(CO): Not much training, just the form college training.

(Interviewer): How familiar are you with the World Health Organization Guidelines for rehydration therapy for diarrheal illnesses?

(CO): Yer am aware.

(Interviewer): How much training have you had regarding these guidelines?

(CO): The IMCI part only in college.

(Interviewer): How do you think a child’s family would react if you wanted to use an NG tube for rehydrating their child?

(CO): Yer thy feel bad because they don’t know what you are doing, at fast before doing that I think the best thing to do is telling the parents what you are doing but for them they feel not comfortable. But if they know what you are doing they will appreciate.

(Interviewer): So are there any cultural beliefs that hinder from the usage of the NG tubes

(CO): I have not heard of any, but maybe IV lines they have beliefs with them that maybe if you put the child would die.

(Interviewer): So what are the biggest barriers you face while rehydrating children?

(CO): The biggest barrier is when you have a child with some dehydration and you have to give ORS but to some point the child drink. So you are supposed to calculate now how much fluid you are supposed to give IV so it’s supposed to plan B but now you go to plan C, because the child isn’t able to take the ORS you have given as per the time you have given, so calculating and how much you can put now instead of the 75MLS.

(Interviewer): And you also mentioned availability of resources.

(CO): Supplies sometimes, the fluid is not there the child might have malnutrition and you are supposed to give the fluid and it’s not there.

(Interviewer): So according to your opinion do you think NGs are as good as IV?

(CO): I don’t think so; I think IV is the best if you get them.

(Interviewer): Okay, so that’s the end of the interview and the survey. Thank you.

(CO): Thank you.

**INTERVIEW #10 AT A REFARAL HOSPITAL.**

(Interviewer): You have gone through a scenario about resuscitating a child with dehydration. When you think about treating dehydration, what are the first methods that come to mind?

(CO): Using IV fluids, oral rehydration.

(Interviewer): What options do you consider when there are difficulties?

(CO): Like I said I will consider using an NGT AND oral rehydration if the kid can take it orally.

(Interviewer): What improves your ability to provide rehydration for children? (CO): Having access to those good IV fluids like resommal, yer those kinds of fluids.

(Interviewer): In the case scenario, you suggested using an NG tubes for rehydration, what were your reasons for these?

(CO): Okay in that question you said that an IV cannula is hard to put and giving with an NGT will be the best option. It would be more efficient.

(Interviewer): Tell me about circumstances in which you would use a nasogastric tube for rehydration therapy.

(CO): In a patient who is severely dehydrated and I can’t get a ……… to fix an IV cannula.

(Interviewer): What would make it difficult to use an NG tube?

(CO): It wouldn’t be hard to use an NG tube unless patient other conditions, maybe obstruction that will make the NGT not go through but it’s not hard to use an NGT .

(Interviewer): What would make this process easier?

(CO): Just having everything that you need, like you lubricate the tubes. Sometimes we don’t have the lubricant so it’s really hard but if you have everything. Everything will be easier.

(Interviewer): Are there circumstances when you would not use a nasogastric tube for rehydration therapy?

(CO): Yes, when am giving oral, when I am using an IV cannula, when the patient is feeding and can taken orally.

(Interviewer): Are NG tubes available in the setting in which you work?

(CO): Yes.

(Interviewer): Are they always available or sometimes?

(C0): Always available.

(Interviewer): What training or experience have you had with using nasogastric tubes?

(CO): I have used quit a lot; I have used NGTS to do gastric lavage to feed babies in NBU.

(Interviewer): But they have not offered ant training?

(CO): Not in this setting just in college

(Interviewer): How familiar are you with the World Health Organization Guidelines for rehydration therapy for diarrheal illnesses?

(CO): Very aware.

(Interviewer): How much training have you had regarding these guidelines?

(CO): Just in college, protocols the way you manage in a hospital. But we haven’t been trained.

(Interviewer): How do you think a child’s family would react if you wanted to use an NG tube for rehydrating their child?

(CO): Mothers don’t like those NGs being inserted in their children.

(Interviewer): What do you think they think about the NG tube, the thing that causes the negativity towards the NG tubes?

(CO): They think it will traumatize the baby ant those organs that it goes through. (Interviewer): When you take consent from the mother and explain what you want to do, are they receptive towards the idea?

(CO): They are receptive, when you tell them the importance and everything they agree.

(Interviewer): So what are your biggest barriers when rehydrating children generally?

(CO): Some children are very dehydrated you can’t fix a line, some parents are not receptive to the idea of having the kids admitted because of dehydration they think it is not something you should admit a kid for.

(Interviewer): Does everybody in this setting know how to put NG tubes and the right sizes? Or do you think training would improve?

(CO): Training would improve because not everyone knows how to use an NG tube and the right sizes.

(Interviewer): Are there any cultural belief that hinder parents from the use of NG tubes?

(CO): No I don’t think so.

(Interviewer): According to your opinion do you think NGs are as good as IVs? (CO): Yer, they rehydrate like IVs.

(Interviewer): So that’s the end of the survey and interview. Thank you.

INTERVIEW #11 CLINICAL OFFICER AT A HEALTH CENTER.

(Interviewer): You have gone through a scenario about resuscitating a child with dehydration. When you think about treating dehydration, what are the first methods that come to mind?

(CO): So you give ORS that would be the first, secondly apart from encouraging the mother to continue breastfeeding you give ORS then if the child is unable to take orally you give IV.

(Interviewer): What options do you consider when there are difficulties?

(CO): You perform an intraosseous.

(Interviewer):What challenges do you face when resuscitating a child with dehydration?

(CO): Challenges is most of the time we don’t have the stock, the drugs, IV fluid all that we are supposed to use.

(Interviewer): What improves your ability to provide rehydration for children? (CO): There should be persistent supply and again personnel, sometimes I could be busy and there are no nurses around me, as I talk there are only two nurses who are in MCH and me alone here so sometimes I would want to be assisted but there is nobody.

(Interviewer): In the case scenario, you suggested using an NG tube for rehydration therapy, what we your reasons for these?

(CO): Because instead of struggling I could have at least done something to assist this child so meanwhile as I struggle I should assure that I am doing something to help the child.

(Interviewer): Tell me about circumstances in which you would use a nasogastric tube for rehydration therapy.

(CO): In a scenario where the child is severely dehydrated and I am not able to get an IV access

(Interviewer): What would make it difficult to use an NG tube?

(CO): Maybe to get the right size of the NG tube and basically that could be then only alternative.

(Interviewer): What would make this process easier?

(CO): Again I come to the supplies, you should at least have everything in place .

(Interviewer): Are there circumstances when you would not use a nasogastric tube for rehydration therapy?

(CO): That one I don’t remember.

(Interviewer): Are NG tubes available in the setting in which you work?

(CO): Nothing, they are not available.

(Interviewer): What training or experience have you had with using nasogastric tubes?

(CO): If you are not careful sometimes you can get it into the wrong place, you can get into the lungs and also sometimes you need to be very careful so that you can make sure that you are in.

(Interviewer): So have they offered you any training or just the one you had in college ?

(CO): I think I had training in college and I specialized in pediatrics . From then I have not gotten any training.

(Interviewer): How familiar are you with the World Health Organization Guidelines for rehydration therapy for diarrheal illnesses?

(CO):I think am well familiar with them.

(Interviewer): How much training have you had regarding these guidelines?

(CO): The one I got in college and I think I have not gotten any update, and also I have read it through the internet.

(Interviewer): So personal initiative.

(CO): Yes.

(Interviewer): How do you think a child’s family would react if you wanted to use an NG tube for rehydrating their child?

(CO): Well it depends on how I explain to them, sometimes they might react badly but if they are assured that what I am doing is right then the perception will change.

(Interviewer): So what are the biggest barriers you face while rehydrating children?

(C0): The problem is that sometimes they come when its late and am not able to do much.

(Interviewer): So do you think training would improve on rehydration of children?

(CO): Very much.

(Interviewer): Are there any cultural beliefs that hinder parents from putting their child NG tubes?

(CO): Other do believe that it will worsen the situation, so they say no. (Interviewer): So educating the society will improve, so do you think NGs are as good as IVs?

(CO): Yer, its only that it may take time to respond but I think they are as good as IV.

(Interviewer): so that’s the end of our interview and survey. Thank you.

(CO): Thank you.

INTERVIEW 12 NURSE AT A HEALTH CENTER

(Interviewer): You have gone through a scenario about resuscitating a child with dehydration. When you think about treating dehydration, what are the first methods that come to mind?

(Nurse): First line is to give drip and normal saline to rehydrate that’s the most important thing before we find the cause of the diarrhea and then do some investigation check the stool and for malaria.

(Interviewer): What options do you consider when there are difficulties?

(Nurse): Difficulties most of the time is to get that line because they come when they are collapsed, so we can try scalp, if we have a clinical officer around we can do cut down .

(Interviewer): What challenges do you face when resuscitating a child with dehydration? (Nurse): Sometimes you don’t have the IV fluids you might not even have a brannula or giving set.

(Interviewer): What improves your ability to provide rehydration for children?

(Nurse): It’s just to have the supplies and the knowledge and then personnel.

(Interviewer): In the case scenario, you did not suggest using a nasogastric tube for rehydration therapy. What were your reasons for this?

(Nurse): You know fixing a nasogastric tube with that dehydration state sometimes you might go wrong, because as you pass the nasogastric you are supposed to tell the child to swallow.

(Interviewer): Tell me about circumstances in which you would use a nasogastric tube for rehydration therapy.

(Nurse): Maybe if the IV has failed .

(Interviewer): What would make it difficult to use an NG tube?

(Nurse): When the child is lethargic.

(Interviewer): What would make this process easier?

(Nurse): It depends on the state of the child.

(Interviewer): Are there circumstances when you would not use a nasogastric tube for rehydration therapy?

(Nurse): If you can manage to use IV

(Interviewer): Are NG tubes available in the setting in which you work?

(Nurse): No.

(Interviewer): What training or experience have you had with using nasogastric tubes? (Nurse): Nasogastric I think I used them a long time ago, but here they are not available and now because we have a referral nearby there is no need of struggling with this child.

(Interviewer): How familiar are you with the World Health Organization Guidelines for rehydration therapy for diarrheal illnesses?

(Nurse): Its long since I had an update.

(Interviewer): How do you think a child’s family would react if you wanted to use an NG tube for rehydrating their child?

(Nurse): Mostly like mothers they will even run away and when they see that they just say the child is now dying. And the community out here fear such things.

(Interviewer): So do you think educating the community at large would improve anything?

(Nurse): Health education is also important because when you will explain why you are doing that and because they want the child to survive they will accept it.

(Interviewer): So what are the biggest barriers you face while rehydrating children? (Nurse): Not as much because when someone has come to the hospital they will accept what you have to do.

(Interviewer): So availability of the NG tubes is also a barrier?

(Nurse): It’s a barrier because we don’t have them and being in a health center we have not been using them as such.

(Interviewer): So according to your opinion do you think NGs are as good as IVs?

(Nurse): I think IVs are better because the NGs sometimes let’s say you are using milk, you get the tubes have clots hygienically again it can cause the child to diarrhea.

(Interviewer): So that’s the end of the interview and survey. Thank you.

(Nurse): Welcome.

INTERVIEW 13 NURSE AT A HEALTH CENTER

(Interviewer): You have gone through a scenario about resuscitating a child with dehydration. When you think about treating dehydration, what are the first methods that come to mind?

(Nurse): Given ORS if it’s at home we have that one which is locally made with salt and sugar.

(Interviewer): What options do you consider when there are difficulties?

(Nurse): Just referral.

(Interviewer): What challenges do you face when resuscitating a child with dehydration? (Nurse): Like now putting a line in a small kid is not easy, most cases when they require IV is not easy cause maybe the veins have collapsed.

(Interviewer): What improves your ability to provide rehydration for children?

(Nurse): Maybe if we are equipped with all the necessities, whatever is required for resuscitating.

(Interviewer): In the case scenario, you did not suggest using a nasogastric tube for rehydration therapy. What were your reasons for this?

(Nurse): Because we don’t have it, but if we are fully equipped I would have mentioned that but now that we don’t have it I can’t talk about it.

(Interviewer): Tell me about circumstances in which you would use a nasogastric tube for rehydration therapy.

(Nurse): Even in that case of severe dehydration before I refer this patient I van also put an NG tube do something before I refer.

(Interviewer): What would make it difficult to use an NG tube?

(Nurse): Because I am not fully equipped.

(Interviewer): What would make this process easier?

(Nurse): IF an fully equipped, if everything is there then it would be easy for me to do something.

(Interviewer): Are there circumstances when you would not use a nasogastric tube for rehydration therapy?

(Nurse): In cases where I have gotten the IV line I would not use an NG tube or if the child can take orally.

(Interviewer): Are NG tubes available in the setting in which you work?

(Nurse): No.

(Interviewer): What training or experience have you had with using nasogastric tubes? (Nurse): So far no, I remember putting one lastly when I was in college 25years ago but since I qualified nothing.

(Interviewer): How familiar are you with the World Health Organization Guidelines for rehydration therapy for diarrheal illnesses?

(Nurse): I don’t think I have the update.

(Interviewer): How much training have you had regarding these guidelines?

(Nurse): So far none.

(Interviewer): How do you think a child’s family would react if you wanted to use an NG tube for rehydrating their child?

(Nurse): With a good explanation I think they will be positive about it.

(Interviewer): The biggest barriers you face while rehydrating children are?

(Nurse): Availability.

(Interviewer): So do you think training would improve in the management of dehydration for children?

(Nurse): Yer 100%

(Interviewer): So do you think NGs are as good as IVs?

(Nurse): IVs are good, but if you can use the NG before going to the IV it would be better.

(Interviewer): So that’s the end of the interview and survey. Thank you.

**INTERVIEW 14 CLINICAL OFFICER AT A DISTRICT HOSPITAL**

(Interviewer): You have gone through a scenario about resuscitating a child with dehydration. When you think about treating dehydration, what are the first methods that come to mind?

(CO): What basically you need to do, you need to access the type of dehydration you need to know whether it is no dehydration, severe dehydration or severe dehydration then you treat accordingly, if there is no dehydration let them carry ORS at home and encourage them to take additional fluids and if over 6 months you can encourage uji and other foods then encourage breastfeeding, then for some dehydration you can just give them according to the weight ORS that is 75mls per kilo and you can give them somewhere we have an ORT corner where they can be given that ORS for the next 4 hours.

(Interviewer): What options do you consider when there are difficulties?

(CO): When looking for a line becomes a challenge you can still call for colleagues around for teamwork we can even conduct the child to see the medical officer we review together then we share then we see the next course of option that is referral technically if its fixing a line we might succeed.

(Interviewer): What challenges do you face when resuscitating a child with dehydration? (CO): Basically maybe when you miss a line cause when you access a line you start giving the fluids.

(Interviewer): What improves your ability to provide rehydration for children?

(CO): Increasing supply, making sure the emergency tray is ready , like ringers lactate now provision is not so frequent so we make sure we secure it from outside and we make sure our ORT is working.

(Interviewer): In the case scenario, you did not suggest using a nasogastric tube for rehydration therapy. What were your reasons for this?

(CO): Basically also nasogastric tube is good…………..

(Interviewer): Tell me about circumstances in which you would use a nasogastric tube for rehydration therapy.

(CO): Incase where we have malnutrition, or in severe dehydration where we are not able to get a line.

(Interviewer):What would make it difficult to use an NG tube?

(CO): I think it’s not difficult.

(Interviewer): What would make this process easier?

(CO): It depends with the technical knowhow and availability of those resources.

(Interviewer): Are there circumstances when you would not use a nasogastric tube for rehydration therapy?

(CO): Maybe issues of comatose patients.

(Interviewer): Are NG tubes available in the setting in which you work?

(CO): Currently we don’t have NG tubes.

(Interviewer): What training or experience have you had with using nasogastric tubes? (CO): We have used in resuscitation

(Interviewer): How familiar are you with the World Health Organization Guidelines for rehydration therapy for diarrheal illnesses?

(CO): I am familiar with it.

(Interviewer): How much training have you had regarding these guidelines?

(CO): Fairly some few.

(Interviewer): How do you think a child’s family would react if you wanted to use an NG tube for rehydrating their child?

(CO): They might be looking worried and they see the child is very sick but when you talk to them they are assured.

(Interviewer): So do you think training would improve the use of NG tubes?

(CO): Yer, like you see like right now medicine is dynamic I think we need to get updated.

(Interviewer): so do you think NGs are as good as IV?

(CO): They are especially like now we have administered ORS which is very good. (Interviewer): So that’s the end of the interview thank you.

INTERVIEW 15 COMMUNITY HEALTH WORKER AT A DISTRICT HOSPITAL

(Interviewer): You have gone through a scenario about resuscitating a child with dehydration. When you think about treating dehydration, what are the first methods that come to mind?

(CHW): Boiling water add salt with a little sugar then after boiling let it come to room temperature.

(Interviewer): What options do you consider when there are difficulties?

(CHW): You sent to the nearest hospital.

(Interviewer): What challenges do you face when resuscitating a child with dehydration? (CHW): Availability of resources.

(Interviewer): What improves your ability to provide rehydration for children?

(CHW): We teach mothers that when they see the signs of dehydration they take their children to the nearest hospital.

(Interviewer): In the case scenario, you did not suggest using a nasogastric tube for rehydration therapy. What were your reasons for this?

(CHW): I have not experienced such.

(Interviewer): Tell me about circumstances in which you would use a nasogastric tube for rehydration therapy.

(CHW): When the child has the hole between the teeth we are unable to use it.

(Interviewer): Are there circumstances when you would not use a nasogastric tube for rehydration therapy?

(CHW): Especially here we don’t have the knowledge so we are inexperienced.

(Interviewer): Are NG tubes available in the setting in which you work?

(CHW): They are available.

(Interviewer): All the time.

(CHW): Yer, but you know here most of the time we refer them.

(Interviewer): What training or experience have you had with using nasogastric tubes? (CHW): None.

(Interviewer): How familiar are you with the World Health Organization Guidelines for rehydration therapy for diarrheal illnesses?

(CHW): I am familiar.

(Interviewer): How much training have you had regarding these guidelines?

(CHW): None.

(Interviewer): How do you think a child’s family would react if you wanted to use an NG tube for rehydrating their child?

(CHW): They will take it strange but it’s better to teach them.

(Interviewer): So what are the biggest barriers you face while rehydrating children? (CHW): We lack knowledge.

(Interviewer): So you think training would improve?

(CHW): Yes.

(Interviewer): do you think NGs are as good as IV?

(CHW): So that’s the end of the interview and survey. Thank you.

**INTERVIEW 16 CLINICAL OFFICER AT A HEALTH CENTER**

(Interviewer): You have gone through a scenario about resuscitating a child with dehydration. When you think about treating dehydration, what are the first methods that come to mind?

(CO): Maybe by ORS and then IV fluids.

(Interviewer): What options do you consider when there are difficulties?

(CO): I will have to refer.

(Interviewer): What challenges do you face when resuscitating a child with dehydration? (CO): The first is if we start with the mum of the kids they don’t understand what is happening and sometimes if you want to look for IV and you want to shave they don’t want that and sometimes we don’t have the IV sets for these small kids so sometimes I just have to approximate.

(Interviewer): So it’s the mother’s attitude and availability.

(CO): Yes

(Interviewer): What improves your ability to provide rehydration for children?

(CO): I think we have to first sensitize the mothers so that at least they can be able to bring children as early as possible and again in the medical facility as the medical personnel I think not all of us are trained, if am not around someone comes with dehydration they can’t get a line they refer. So I think each health personnel should be trained on how to identify the signs and symptoms of dehydration

(Interviewer): In the case scenario, you suggested using a nasogastric for rehydration therapy what was your reason for that?

(CO): Because now if we do not rehydrate this child, she is going to die so the only option is to put an NG tube so from there you can be able to get the vein and then you change. Just as an emergency.

(Interviewer): Tell me about circumstances in which you would use a nasogastric tube for rehydration therapy.

(CO): So you get this child and you can’t get the vein and you get this child who is severely dehydrated or is vomiting everything so you have to use a nasogastric tube. (Interviewer): What would make it difficult to use an NG tube?

(CO): The age of the child sometimes you know the child maybe too small and the NG tube that you have is the larger size and again not all people know how to put an NG tube because sometimes you might end up getting into the lungs so at least somebody has to be trained on that. (Interviewer): What would make this process easier?

(CO): The process would be easier if I mean now like the NG tube and everything is available the right size I mean all the sizes because you can’t know the kind of kid you are going to get and again the personnel should also be trained each and everybody at least. And again the attitude of the parents because when they see you inserting the NG tube they are like the child is dying so as the medical personnel we should inform the mother of what you are going to do so that the mother cooperates.

(Interviewer): Are there circumstances when you would not use a nasogastric tube for rehydration therapy?

(CO): If the patient has upper respiratory tract infection or has a tumor or something so there is no way you can insert that tube

(Interviewer): Are NG tubes available in the setting in which you work?

(CO): Yer they are there, the larger sizes but the small ones are not there.

(Interviewer): What training or experience have you had with using nasogastric tubes? (CO): It just covers the normal routine procedures, and I got the experience while I was in MTRH.

(Interviewer): So they have not offered any training?

(CO): No, There is no training about that.

(Interviewer): How familiar are you with the World Health Organization Guidelines for rehydration therapy for diarrheal illnesses?

(CO): I am familiar.

(Interviewer): How much training have you had regarding these guidelines?

(CO): None just the one we got from school and from experience

(Interviewer): How do you think a child’s family would react if you wanted to use an NG tube for rehydrating their child?

(CO): Of course they won’t like it, the first thing they think is that the patient is that the patient is dying so they might oppose that but as a clinician you have to seat with them and tell them what you are going to do and what might happen if you don’t use that, so that they may be able to know what’s going on.

(Interviewer): So what are the biggest barriers you face while rehydrating children? (CO): Availability of the right fluids, sometimes I might need to use Hartman’s and I don’t have it, maybe I have only normal saline and the best fluid for dehydration is Hartmann’s and again the IV sets I don’t have for the children now I have to approximate which is not easy sometimes you may end up over rehydrating or under rehydrate because you fear you are going to over rehydrate.

(Interviewer): So do you think everyone knows how to put an NG tube and the right size?

(CO): No.

(Interviewer): So do you think training would improve?

(CO): Yeah, if people were trained about that it would be a good improvement. (Interviewer): So according to your opinion do you think NG tubes are as good as IV? (CO): They are not that good as IV lines but sometimes.

(Interviewer): So that’s the end of the interview and the survey. Thank you.

INTERVIEW 17 NURSE AT A REFFARAL HOSPITTAL.

(Interviewer): You have gone through a scenario about resuscitating a child with dehydration. When you think about treating dehydration, what are the first methods that come to mind?

(Nurse): Okay you are looking at venous prolapse and shock and then you are looking at hypothermia and hypoglycemia. So those are the first things that come on my mind.

(Interviewer): What options do you consider when there are difficulties?

(Nurse): I would seek further treatment like maybe I consult

(Interviewer): What challenges do you face when resuscitating a child with dehydration?(Nurse): Sometimes the history may not come out clear as to the cause of the dehydration and sometimes you find maybe the mother has given herbs. Or a child has come from another facility and the veins have been tampered with.

(Interviewer): What improves your ability to provide rehydration for children?

(Nurse): Maybe through experience I would say trying to fix a line or another method hydrate orally through an NG tube.

(Interviewer): In the case scenario, you did not suggest using a nasogastric tube for rehydration therapy. What were your reasons for this?

(Nurse): First you need to consider the sugars when a child comes with dehydration so the sugars will guide you on which type of fluids to give, so you consider will the NG tube help so I might want to give normal saline via nasogastric or ringers so before I go on that I have to give oral.

(Interviewer): Tell me about circumstances in which you would use a nasogastric tube for rehydration therapy.

(Nurse): If I fail to get a line and also the child is not able to take orally maybe has another condition and might not be able to swallow.

(Interviewer): What would make it difficult to use an NG tube?

(Nurse): Maybe if the child has any other nasal condition like nasal polyps or difficulty in breathing.

(Interviewer): What would make this process easier?

(Nurse): First you need to rule out if the airway is clear and the child is not in distress then I will go for the NG tube.

(Interviewer): Are there circumstances when you would not use a nasogastric tube for rehydration therapy?

(Nurse): Okay, if the child is in shock what I want is to reverse the shock so I will not use the NG tube

(Interviewer): Are NG tubes available in the setting in which you work?

(Nurse): Yes they are available.

(Interviewer): All the time?

(Nurse): Yes.

(Interviewer): What training or experience have you had with using nasogastric tubes? (Nurse): I was trained as a nurse, so I have my experience through training at college, so while working I have gotten then experience.

(Interviewer): How familiar are you with the World Health Organization Guidelines for rehydration therapy for diarrheal illnesses?

(Nurse): Okay, these are guidelines we use every dayso am quite familiar with them. (Interviewer): How much training have you had regarding these guidelines?

(Nurse): I think it’s on going because it’s something we are dealing with every day in our setup.

(Interviewer): But they have not offered you any training?

(Nurse): We have had the continuous medical education.

(Interviewer): How do you think a child’s family would react if you wanted to use an NG tube for rehydrating their child?

(Nurse): Most mothers what they have on their mind is that their child is dying is that their child is dying so there is no hope so they are doing their last resort.

(Interviewer): What are the biggest barriers to rehydrating children?

(Nurse):It’s just getting a line, how to access a line especially when they come in late it’s a barrier to us. But if they come early or they are referred early from the other facility then you will be able to access a line.

(Interviewer): So do you think everybody knows how to put NG tubes the right sizes? (Nurse): Most of the people here are trained so they know the right sizes to put. (Interviewer): In rehydration according to your opinion do you think NGs are as good as IVs?

(Nurse): It depends on the condition.

(Interviewer): That’s the end of the interview and the survey. Thank you.

(Nurse): Thank you too.

INTERVIEW 18 MEDICAL OFFICER AT E REFFARAL HOSPITAL.

(Interviewer): You have gone through a scenario about resuscitating a child with dehydration. When you think about treating dehydration, what are the first methods that come to mind?

(MO): You need ringers lactate and then you give according to the weight 30mls/kg for the fast half hour then 70mls/kg for the next two and a half hours.

(Interviewer): What options do you consider when there are difficulties?

(MO): You can consider ORS if the child is awake or you fix an intraosseous line.

(Interviewer): What challenges do you face when resuscitating a child with dehydration? (MO): The main problem is line.

(Interviewer): What improves your ability to provide rehydration for children?

(MO): I think its experience.

(Interviewer): In the case scenario, you did not suggest using a nasogastric tube for rehydration therapy. What were your reasons for this?

(MO): It’s not a first line but you can use it as a last option most of the time you can use an intraosseous line.

(Interviewer): Tell me about circumstances in which you would use a nasogastric tube for rehydration therapy.

(MO): Especially when a child is malnourished you need nasogastric tube to give ressomal.

(Interviewer): What would make it difficult to use an NG tube?

(MO): There is no difficultly, just that we don’t practice it as much.

(Interviewer): What would make this process easier?

(MO): To integrate it.

(Interviewer): Are there circumstances when you would not use a nasogastric tube for rehydration therapy?

(MO): Sometimes when you have congenital anomalies causing obstruction like nasophargeal fistula.

(Interviewer): Are NG tubes available in the setting in which you work?

(MO): Yes.

(Interviewer): All the time?

(MO): All the time.

(Interviewer): All the sizes?

(MO): All the sizes.

(Interviewer): What training or experience have you had with using nasogastric tubes? (MO): Not much cause most of the time it’s the nurses who fix them, so we don’t get an opportunity to fix them. But sometimes we do, but personally I don’t like fixing them. (Interviewer): so have they offered you any training on the usage of NG tubes?

(MO): No, but I guess that will be personal initiative, you can just learn in the job.

(Interviewer): How familiar are you with the World Health Organization Guidelines for rehydration therapy for diarrheal illnesses?

(MO): Very aware.

(Interviewer): How much training have you had regarding these guidelines?

(MO): Training…………………. EPL.

(Interviewer): How do you think a child’s family would react if you wanted to use an NG tube for rehydrating their child?

(MO): Most of the time they are receptive as long as you explain to them you are going to help their child, I have never gotten a parent who refuses NG tube.

(Interviewer): What are the biggest barriers you face in this setting while rehydrating children?

(MO): Sometimes they can come when they are in shock so getting a line becomes a problem then we don’t do so much of the intraosseous

(Interviewer): So according to your opinion do you think NGs are as good as IV while rehydrating children?

(MO): I don’t think they are as effective cause it will depend on absorption especially if the child has diarrhea the it will have absorption problem so it’s not as effective but it’s still an option the last option.

(Interviewer): So that’s the end of the survey and interview. Thank you very much. (MO): Okay.

INTERVIEW 19 CLINICAL OFFICER AT A REFFARAL HOSPITAL.

(Interviewer): You have gone through a scenario about resuscitating a child with dehydration. When you think about treating dehydration, what are the first methods that come to mind?

(CO): I think of the cause of dehydration, I think that’s the main thing.

(Interviewer): What options do you consider when there are difficulties?

(CO): First few minutes you don’t waste time looking for an IV access you can think of an intraosseous trying to rehydrate the child fast until you are able to get the IV cause intarosseous can last for not more than 8 hours

(Interviewer): What challenges do you face when resuscitating a child with dehydration?

(CO): Challage is not getting a line another thing is you would like to train more people on using an intraosseous cause not all clinicians know how to fix an inraosseous

(Interviewer): What improves your ability to provide rehydration for children?

(CO): More training and availing the required pack of management.

(Interviewer):In the case scenario, you did not suggest using a nasogastric tube for rehydration therapy. What were your reasons for this?

(CO): Nasogastric tube is okay when the child is not vomiting , when you put an NG tube when a child is not vomiting it saves you when you are still looking for the line but when the child is vomiting you wouldn’t want to risk cause sometimes you are fearing of vomiting again aspiration.

(Interviewer): Tell me about circumstances in which you would use a nasogastric tube for rehydration therapy.

(CO): You can use a nasogastric tube when the child is not vomiting.

(Interviewer):What would make it difficult to use an NG tube?

(CO): Actually its not difficult to use an NG tube but actually when you put an NG tube when a child is vomiting there is nothing you are doing.

(Interviewer): What would make this process easier?

(CO): Its not a complex procedure.

(Interviewer): Are there circumstances when you would not use a nasogastric tube for rehydration therapy?

(CO): I think we are not going to always use an NG tube.

(Interviewer): Are NG tubes available in the setting in which you work?

(CO): Yes we do have NG tubes but sometimes we don’t have the right features and sometimes we improvise feeding tubes.

(Interviewer): What training or experience have you had with using nasogastric tubes?

(CO): I have never attended a training as per say for NG tube but in my learning in school we had mankins where we would practice on how to put an NG tube, that’s where I learned and also prtactise.

(Interviewer): How familiar are you with the World Health Organization Guidelines for rehydration therapy for diarrheal illnesses?

(CO): I think am familiar.

(Interviewer):How much training have you had regarding these guidelines?

(CO): We always have CMEs updating ourselves onmanagement of several illnesses including dehydration.

(Interviewer): But they have not offered you any training?

(CO):Not really we only update ourselves.

(Interviewer): How do you think a child’s family would react if you wanted to use an NG tube for rehydrating their child?

(CO): They are not always cooperative they think if you want to insert an NG tube this must be a very sick child, actually before you insert an NG tube you have to tell them why you are doing this and to reassure them there is no harm.

(Interviewer): What are the biggest barriers you face while rehydrating children.

(CO): The biggest challenge I have faced personally is traditional rules. A mother does come and says we were told that we a child becomes sick no injections some cultures and religion so you get a bit challenged and sometimes they would want to resist physically, they would want to fight and ask you to only give oral medicine which is hard.

(Interviewer): So do you think training would improve much on rehydrating children. (CO): Yer training of staff and another thing community awareness.

(Interviewer): So according to your opinion do you think NGs are as good as IV?

(CO): Yer they are good but depending not always, NG tube is not as good as IV my opinion but also in the other hand its good cause in like in fluid overload you rarely get it while using NGs.

(Interviewer): That’s the end of the interview and survey. Thank you.

(CO): You are welcome.

INTERVIEW 20 NURSE AT A HEALTH CENTER.

(Interviewer): You have gone through a scenario about resuscitating a child with dehydration. When you think about treating dehydration, what are the first methods that come to mind?

(CO):We encourage ORS then we observe how much the child can take in a certain time and then when we know he can take orally I encourage breastfeeding and frequent feeding.

(Interviewer): What options do you consider when there are difficulties?

(CO): We don’t hesitate we just refer.

(Interviewer): What challenges do you face when resuscitating a child with dehydration?

(Nurse): There are many challenges one of them is putting a line and failing and another one is refusal of referral, when you refer the client the most thing that you will get is that they don’t have the money for referral, so we refer the next place like district.

(Interviewer): What improves your ability to provide rehydration for children?

(Nurse): Personnel and then enough rehydration fluids

(Interviewer): In the case scenario, you did not suggest using a nasogastric tube for rehydration therapy. What were your reasons for this?

(Nurse): Most of the time I don’t do it myself because one the status of the child, if the child is positive I don’t use that because of the nasal mucosa you might puncture it

(Interviewer): Tell me about circumstances in which you would use a nasogastric tube for rehydration therapy.

(Nurse):If the child is vomiting a lot and I know the status and is very dehydrated where I cannot get the line.

(Interviewer): What would make it difficult to use an NG tube?

(Nurse): The status we are discouraging not to use.

(Interviewer): What would make this process easier?

(Nurse): That one I don’t know.

(Interviewer): Are there circumstances when you would not use a nasogastric tube for rehydration therapy?

(Nurse): If parents they don’t complain they don’t like.

(Interviewer): Are NG tubes available in the setting in which you work?

(Nurse): Yes we have.

(Interviewer): All the time?

(Nurse): Yer, we get the supply from KEMSA, like the kit we recently received had nasogastric tubes.

(Interviewer): What training or experience have you had with using nasogastric tubes?

(Nurse): The last training I had was when I was in school, in 2009.

(Interviewer): How familiar are you with the World Health Organization Guidelines for rehydration therapy for diarrheal illnesses?

(Nurse): I haven’t read a lot but I can’t miss.

(Interviewer): How much training have you had regarding these guidelines?

(Nurse): I have not had any only that I learned in school.

(Interviewer): How do you think a child’s family would react if you wanted to use an NG tube for rehydrating their child?

(Nurse):Before I do anything I have to consult the family, to consult the person that has come with the child the parent or the guardian then you counsel them abit about the nasogastric tube, the advantage of putting the child on the nasogastric tube and you also give the disadvantage you don’t only you don’t only give one side. So if they agree you fix immediately if they don’t then they go ahead. But if they refuse you just refer them to the district hospital.

(Interviewer): What are the biggest barriers you face while rehydrating children? (Nurse): We have never gotten such a dehydrated so much that we might be getting the barriers we had one but we referred.

(Interviewer): So do you think according to your opinion NGs are as good as IV?

(Nurse): Not 99%.

(Interviewer): So that’s the end of the interview and survey. Thank you.

(Nurse): You are welcomed.

INTERVIEW 21 NURSE AT A SUBDISTRICT HOSPITAL.

(Interviewer): You have gone through a scenario about resuscitating a child with dehydration. When you think about treating dehydration, what are the first methods that come to mind?

(Nurse):First method is the oral method.

(Interviewer): What options do you consider when there are difficulties?

(Nurse): Repeat first difficulty in which sense.

(Interviewer):like you are resuscitating a child for example with severe dehydration the child can’t take orally, can’t retain and the line has failed like in our case what would you consider doing?

(Nurse): If it fails completely that’s an admission, once admitted at my level as a nurse officer we are supposed to call a pediatricianto give a more specialized treatment at our level.

(Interviewer): What challenges do you face when resuscitating a child with dehydration?

(Nurse): In this set up mostly like in our case we don’t have enough man power in a specialized way that is the pediatrician we don’t have them. We have a pedestrian who is a clinical officer and her label may not be as much as the consultant pediatricianso that’s one aspect second aspect we do not have a ward for admission though it is a district hospital but it’s a newly created district hospital without proper facilitating set up so space for a ward for admission to carry out specialized treatment might not be there so we face the challenge that we must now refer this patient to MTRH for further management so in a way it incurs financial management cause there must be an ambulance.

(Interviewer): What improves your ability to provide rehydration for children?

(Nurse): First is training of the man power and then creation of a small room for rehydration purposes and have a proper ward for pediatrics.

(Interviewer): In the case scenario, you did not suggest using a nasogastric tube for rehydration therapy. What were your reasons for this?

(Nurse): I may have not suggested it as part of it but my experience has had some bad episodes of that one, sometimes you insert those nasogastric tubes and then in the process you find it has gone to the lungs, so that one tells me if at my level if I continue doing that because mine is more administrative and you realize this other people if they don’t have that kind of experience they may go into the lungs without knowing. So it needs more attention for updates of the staff so that at least when they are doing it, they know exactly what they are doing otherwise I have seen even myself doctors who do those things they put in the lungs and when we are advising them and they are insisting no they are in the stomach. So am afraid because of the past experiences.

(Interviewer): Tell me about circumstances in which you would use a nasogastric tube for rehydration therapy

(Nurse): I will give it to mostly children around 3 years those ones I feel much more comfortable than in very small ones.

(Interviewer):What would make it difficult to use an NG tube?

(Nurse): it’s not difficult actually, it’s the professional aspect of it the technical aspect that people have to be quite conversant with it before using, most kids will just die before you realize that you have put this things in the wrong place.

(Interviewer): What would make this process easier?

(Nurse): The easier way line now in our set up is once we create the ward and then we need to educate our own staff, give them the most recent update on the same, once they get that one is one of the best way to rehydrate the child without even going to the IVs

(Interviewer): Are there circumstances when you would not use a nasogastric tube for rehydration therapy?

(Nurse): I don’t think it’s there. There is none.

(Interviewer): Are NG tubes available in the setting in which you work?

(Nurse): They are there.

(Interviewer): All the time they are there?

(Nurse): They are there.

(Interviewer): What training or experience have you had with using nasogastric tubes? (Nurse): The experience is just that the nasogastric needs first is the hygiene part of it you must at them also for expiring date and then you have to look at the age and then the sizes of the nasogastric tubes and then you must have to also see that they have marks that show that this is the extent that it has to go that if you go beyond there then you are perforating.

(Interviewer): So have they offered you any training regarding the usage of nasogastric tubes?

(Nurse): I trained as a younger nurse but any other training no.

(Interviewer): How familiar are you with the World Health Organization Guidelines for rehydration therapy for diarrheal illnesses?

(Nurse): The familiarity that I have is maybe understanding from the guide lines that I have about IMCI, because they have been training people from different places and when they come here and they live you with some guide lines I have been going through, so I think I have the standard guidelines of the IMCI and through that I think I have the grasp of the most recent information.

(Interviewer): How do you think a child’s family would react if you wanted to use an NG tube for rehydrating their child?

(Nurse): Particularly families which are well up they have no problem but families which are slightly illiterate they think that once it has been put its over fort the child so they have that negative attitude that the NG tube.

(Interviewer): So what are the biggest barriers you face while rehydrating children. (Nurse): The biggest barrier we have here is space.

(Interviewer): Do you think training would improve people’s ability on the usage of NG tubes?

(Nurse): Very well.

(Interviewer): So according to your opinion do you think NG tubes are as good as IV? (Nurse): Infarct to me if they are well adopted they are better than IV.

(Interviewer): So that’s the end of the interview and the survey. Thank you.

INTERVIEW 22 COMMUNITY HEALTH WORKER AT A SUBDISTRICT HOSPITAL.

(Interviewer): You have gone through a scenario about resuscitating a child with dehydration. When you think about treating dehydration, what are the first methods that come to mind?

(CHW): First of all you give ORS,

(Interviewer): What options do you consider when there are difficulties?

(CHW): Either you refer or you can give antibiotics at the same time.

(Interviewer): What challenges do you face when resuscitating a child with dehydration? (CHW): The issue about not finding the vein that one of them, and then you find some mothers are very difficult to deal with.

(Interviewer): What improves your ability to provide rehydration for children?

(CHW): At least teach the mothers hygiene something like that and if something like that happens at home you tell them they can take something like salt and sugar and mix together, year home based care.

(Interviewer): In the case scenario, you did not suggest using a nasogastric tube for rehydration therapy. What were your reasons for this?

(CHW): We don’t have those ones, I cannot lie to you.

(Interviewer): Tell me about circumstances in which you would use a nasogastric tube for rehydration therapy.

(CHW): When there are no veins.

(Interviewer): What would make it difficult to use an NG tube?

(CHW): Sometimes you can find maybe the kid has infection so you can’t use and sometimes the NG tubes are not available.

(Interviewer): What would make this process easier?

(CHW):Unless they make them available.

(Interviewer): Are there circumstances when you would not use a nasogastric tube for rehydration therapy?

(CHW): No.

(Interviewer): Are NG tubes available in the setting in which you work?

(CHW): No.

(Interviewer): What training or experience have you had with using nasogastric tubes?

(CHW): None.

(Interviewer): How familiar are you with the World Health Organization Guidelines for rehydration therapy for diarrheal illnesses?

(CHW): Not that familiar.

(Interviewer): How much training have you had regarding these guidelines?

(CHW): None.

(Interviewer): How do you think a child’s family would react if you wanted to use an NG tube for rehydrating their child?

(CHW): You have to counsel them first before you put the NG tube.

(Interviewer): So after counseling they will be receptive?

(CHW): It depends some will be receptive some defensive.

(Interviewer): So what are the biggest barriers you face in this setting while rehydrating children?

(CHW): I think it’s just lack of those things.

(Interviewer): Do you think further training would improve the use of NG tubes in this setting?

(CHW): Mmmh!

(Interviewer): So according to your opinion do you think NG tubes are as good as IV? (CHW): they are easy to manage.

(Interviewer): So you think they are as good as IV?

(CHW): Yes.

(Interviewer): Thank you that’s the end of the survey and the interview.

INTERVIEW 23 AT A SUBDISTRICT HOSPITAL.

(Interviewer): You have gone through a scenario about resuscitating a child with dehydration. When you think about treating dehydration, what are the first methods that come to mind?

(Nurse):It now depends on the dehydration is it moderate is it severe; it goes with the degree of dehydration.

(Interviewer): What options do you consider when there are difficulties?

(Nurse): When you are in a referral hospital because you will not refer patients somewhere else so the doctor will be there I’ll do a cut down and then the child continues with IV fluid and when you are in a health center you will put the NG tube so after the NG tube you will arrange for the referral.

(Interviewer): What challenges do you face when resuscitating a child with dehydration? (Nurse): first of all let me say there might be one challenge because you might not get a doctor to do the cut down and then it can’t be done in our institution here other challenge is we don’t have an ambulance for referral it was knocked down and then perhaps getting a doctor.

(Interviewer): What improves your ability to provide rehydration for children?

(Nurse): Perhaps updates, u know most of the time they are changes.

(Interviewer): In the case scenario, you did suggested using a nasogastric tube for rehydration therapy. What were your reasons for this?

(Nurse): It’s just to correct the electrolyte imbalance.

(Interviewer): Tell me about circumstances in which you would use a nasogastric tube for rehydration therapy.

(Nurse):One is severe dehydration and perhaps unconscious patient that who cannot take in and in a pre-term baby.

(Interviewer): What would make it difficult to use an NG tube?

(Nurse):Am learned about it so there is nothing difficult.

(Interviewer): Are there circumstances when you would not use a nasogastric tube for rehydration therapy?

(Nurse): Unless it’s moderate you just give orals.

(Interviewer): Are NG tubes available in the setting in which you work?

(Nurse): Yes.

(Interviewer): They are always there.

(Nurse): Yes.

(Interviewer): What training or experience have you had with using nasogastric tubes? (Nurse): Only for IMCI

(Interviewer): How familiar are you with the World Health Organization Guidelines for rehydration therapy for diarrheal illnesses?

(Nurse): I am not sure about it but what I was telling you is training, to be trained again .

(Interviewer): How do you think a child’s family would react if you wanted to use an NG tube for rehydrating their child?

(Nurse): That one you counsel the parent and the family first and then you tell them the importance of using the NG.

(Interviewer): So what are the biggest barriers you face while rehydrating children? (Nurse): …………………………

(Interviewer): Like you said personnel and availability of resources

(Nurse): Only that, but curiously most of the things are there unless you don’t want to do the nursing part of it and mostly people here are ready to work there is no any other barrier I can say unless I create a medical barrier.

(Interviewer): So do you think training would improve on the management of dehydration in children?

(Nurse): Updates yes because they change things and we don’t know, they change management and we don’t know, so if they take us with the updates the trainings it will change.

(Interviewer): So according to your opinion are NG tubes as good as IV?

(Nurse): I think no, but they are good because at least it will rehydrate the child. (Interviewer): So that’s the end of the survey and interview. Thank you.

INTERVIEW 24 NURSE AT A SUBDISTRICT HOSPITAL

(Interviewer): You have gone through a scenario about resuscitating a child with dehydration. When you think about treating dehydration, what are the first methods that come to mind?

(Nurse):ORS the other one is intravenous.

(Interviewer): What options do you consider when there are difficulties?

(Nurse): Call the next on call to assist in fixing the line, and the next level maybe the clinical officer will assist me in writing the referral letter.

(Interviewer): What challenges do you face when resuscitating a child with dehydration? (Nurse): Sometimes the veins are collapsed it’s hard to get the veins especially for pediatricsit’s difficult.

(Interviewer): What improves your ability to provide rehydration for children?

(Nurse): Health education on mothers to proper personal hygiene, proper dieting and maybe proper breastfeeding. The children will be able to gain immunity to resist diarrhea and vomiting.

(Interviewer): In the case scenario, you did not suggest using a nasogastric tube for rehydration therapy. What were your reasons for this?

(Nurse): Nasogastric needs skills.

(Interviewer): Tell me about circumstances in which you would use a nasogastric tube for rehydration therapy.

(Nurse):Coma and in NBU in premature babies.

(Interviewer): What would make it difficult to use an NG tube?

(Nurse): I can fix it it’s just that the set up now doesn’t have enough facility and room.(Interviewer): What would make this process easier?

(Nurse): Just avail the supplies.

(Interviewer): Are there circumstances when you would not use a nasogastric tube for rehydration therapy?

(Nurse): Our level doesn’t have pediatric room and also consultants to consult maybe.

(Interviewer): Are NG tubes available in the setting in which you work?

(Nurse): Yer.

(Interviewer): All the time?

(Nurse): No.

(Interviewer): What training or experience have you had with using nasogastric tubes? (Nurse): My initial one in college.

(Interviewer): How familiar are you with the World Health Organization Guidelines for rehydration therapy for diarrheal illnesses?

(Nurse): I am not familiar.

(Interviewer): How much training have you had regarding these guidelines?

(Nurse): None.

(Interviewer): How do you think a child’s family would react if you wanted to use an NG tube for rehydrating their child?

(Nurse): If the child is seriously ill they will agree.

(Interviewer): So what are the biggest barriers you face in this setting while rehydrating children?

(Nurse): Skills, room then the NG tubes are not always available sometimes they are out of stock.

(Interviewer): So do you think training would improve on the usage of NG tubes and make people more receptive towards them.

(Nurse): Yes.

(Interviewer): So according to your opinion do you think NGs as good as IV?

(Nurse): Because I have not rehydrated a kid by NG I cannot compare.

(Interviewer): So that the end of the interview and the survey Thank you

INTERVIEW 25 NURSE AT A SUBDISTRICT HOSPITAL

(Interviewer): You have gone through a scenario about resuscitating a child with dehydration. When you think about treating dehydration, what are the first methods that come to mind?

(Nurse): Giving ORS and continuation of breastfeeding.

(Interviewer): What options do you consider when there are difficulties?

(Nurse): Maybe a mother is in a set up where she cannot get ORS easily encourage her to give fluids of whatever method it could be fluids it could be porridge.

(Interviewer): What challenges do you face when resuscitating a child with dehydration?

(Nurse): Because we have not actually identified a rehydration center but we have been trying.

(Interviewer): What improves your ability to provide rehydration for children?

(Nurse): The only thing which we need to do is to set it up cause personnel is there.

(Interviewer): In the case scenario, you did not suggest using a nasogastric tube for rehydration therapy. What were your reasons for this?

(Nurse): Its severe dehydration and there is a place where it says the child cannot take orally and I cannot get a vein.

(Interviewer): Tell me about circumstances in which you would use a nasogastric tube for rehydration therapy.

(Nurse): In comatose, in neonates.

(Interviewer): What would make it difficult to use an NG tube?

(Nurse):It’s really not a problem as such as opposed to IV line in fact IV difficult than NG tube.

(Interviewer): What would make this process easier?

(Nurse): Availability of the materials if the NG tube is there ORS and the center is set up then it will pick.

(Interviewer): Are there circumstances when you would not use a nasogastric tube for rehydration therapy?

(Nurse): A child who has maybe severe burns on the face.

(Interviewer): Are NG tubes available in the setting in which you work?

(Nurse): They are available.

(Interviewer): What training or experience have you had with using nasogastric tubes?

(Nurse): Not training as such but I have done control of diarrhealdiseases.

(Interviewer): How familiar are you with the World Health Organization Guidelines for rehydration therapy for diarrheal illnesses?

(Nurse): Just a little on job training but I have not gone for IMCI .

(Interviewer): How much training have you had regarding these guidelines?

(Nurse):I CDC but IMCI no.

(Interviewer): How do you think a child’s family would react if you wanted to use an NG tube for rehydrating their child?

(Nurse): Well I don’t think they will over react if I explain to them.

(Interviewer): So what is the biggest barrier you face in this set up while rehydrating children?

(Nurse): One thing we have not set up a rehydration center but everything is available. (Interviewer): So do you think training would improve on the usage of NG tubes? (Nurse): it would.

(Interviewer): So according to your opinion do you think NGs are as good as IV?

(Nurse): Infarct they are even better. (Interviewer): So that’s the end of the survey and interview. Thank you.

(Nurse): Welcome

INTERVIEW 26 NURSE AT A SUBDISTRICT HOSPITAL.

(Interviewer): You have gone through a scenario about resuscitating a child with dehydration. When you think about treating dehydration, what are the first methods that come to mind?

(Nurse): Fluid replacement, if breastfeeding continue breastfeeding if not breastfeeding gives other foods.

(Interviewer):What options do you consider when there are difficulties?

(Nurse): Options like IV access and admission.

(Interviewer): What challenges do you face when resuscitating a child with dehydration?

(Nurse): Sometimes there are no brannulas the exact fluid for rehydration, you can get one type and dextrose is not good for rehydration.

(Interviewer): What improves your ability to provide rehydration for children?

(Nurse): Maybe more staff to be trained on that IMCI because some of them fixing a line is a problem and provide fluids brannulas and other equipment’s .

(Interviewer): In the case scenario, you suggested using a nasogastric tube for rehydration therapy. What were your reasons for this?

(Nurse): You know the child is very sick so they cannot feed they cannot swallow, nasogastric you know it’s direct to the stomach.

(Interviewer): Tell me about circumstances in which you would use a nasogastric tube for rehydration therapy.

(Nurse): When you don’t get a line, two is when the child cannot drink.

(Interviewer): What would make it difficult to use an NG tube?

(Nurse):it’s not a must you fix and its inside sometimes it fails.

(Interviewer): What would make this process easier?

(Nurse): Maybe just a trained person to fix not everybody can fix a nasogastric tube.

(Interviewer): Are there circumstances when you would not use a nasogastric tube for rehydration therapy?

(Nurse): No, maybe when you get a line it’s not a must you use a nasogastric tube.

(Interviewer): Are NG tubes available in the setting in which you work?

(Nurse): I am not aware I am quite new, I have finished 6 months here.

(Interviewer): What training or experience have you had with using nasogastric tubes?

(Nurse): Its good for rehydration and other purposes maybe when the children are very sick and they are in coma you can use an NG tube.

(Interviewer): So have they offered you any training on the use of NG tubes?

(Nurse): No, the only one I have is the one I had in college.

(Interviewer): How familiar are you with the World Health Organization Guidelines for rehydration therapy for diarrheal illnesses?

(Nurse):I know there is plan A and plan B. plan A you use ORS and you continue breastfeeding and you monitor and you use zinc if there is no improvement after 48 hours you take to the hospital.

(Interviewer):How much training have you had regarding these guidelines?

(Nurse): I don’t have training as much is just that I know a little when you are with the clinicians . I have not been trained it’s just reading and personal initiative .

(Interviewer): How do you think a child’s family would react if you wanted to use an NG tube for rehydrating their child?

(Nurse): It depends on the religion sometimes personal things, some people don’t like. (Interviewer): Even cultural barriers are there?

(Nurse): Yes, we have cultural and personal. Even some refuse oxygen not just nasogastric, you see the baby is gasping but they refuse.

(Interviewer): So you think social education will help?

(Nurse): Yes even social education will help.

(Interviewer): So what are the biggest barriers you face while rehydrating children in this setup?

(Nurse): You know we don’t have a ward so it can fail you can rehydrate with ORS and it fails so you refer, so that’s the problem we are referring patients and dehydration is not something you should refer. So we are referring and sometimes we don’t even have the ambulance like now the ambulance is out of order.

(Interviewer): So according to your opinion do you think NGs are as good as IVs? (Nurse): No because the child can still vomit while using NG tubes but IVs it goes in the system but NG just goes to the stomach so the best is IV.

(Interviewer): So that’s the end of the interview and survey thank you very much.

INTERVIEW 27 CLINICAL OFFICER AT A SUBDISTRICT HOSPITAL.

(Interviewer): You have gone through a scenario about resuscitating a child with dehydration. When you think about treating dehydration, what are the first methods that come to mind?

(CO):Oral.

(Interviewer):What options do you consider when there are difficulties?

(CO): You go for second options like IV.

(Interviewer): What challenges do you face when resuscitating a child with dehydration?

(CO): We don’t have a CCT room, we lack rooms here.

(Interviewer): What improves your ability to provide rehydration for children?

(CO): If we could get a room and staff to manage then that will be very grate.

(Interviewer): In the case scenario, you did not suggest using a nasogastric tube for rehydration therapy. What were your reasons for this?

(CO): I forgot that one am sorry.

(Interviewer): Tell me about circumstances in which you would use a nasogastric tube for rehydration therapy.

(CO): When the two of them fail.

(Interviewer): What would make it difficult to use an NG tube?

(CO): If the child is severely sick and maybe has fits.

(Interviewer): What would make this process easier?

(CO): Okay you try to sedate the child and go for it.

(Interviewer): Are there circumstances when you would not use a nasogastric tube for rehydration therapy?

(CO): Not really

(Interviewer): Are NG tubes available in the setting in which you work?

(CO): Yer.

(Interviewer): All the time?

(CO): No at times.

(Interviewer): What training or experience have you had with using nasogastric tubes?

(CO):Its long since,

(Interviewer): And they have not offered any training

(CO): Yer

(Interviewer): How familiar are you with the World Health Organization Guidelines for rehydration therapy for diarrheal illnesses?

(CO): I have knowledge and I have done the course.

(Interviewer):How much training have you had regarding these guidelines?

(CO): I don’t know, I don’t still remember.

(Interviewer): How do you think a child’s family would react if you wanted to use an NG tube for rehydrating their child?

(CO): It depends on the education.

(Interviewer): So after educating them they become receptive.

(CO): Yer, exactly.

(Interviewer): I this setting what are the biggest barriers you face while rehydrating children?

(CO): Mainly the room.

(Interviewer): So do you think training would improve on the usage of NG tubes?

(CO): Yes.

(Interviewer): So according to your opinion do you think NGs are as good as IV?

(CO): Yer.

(Interviewer): So that’s the end of the interview and survey. Thank you

INTERVIEW 28 CLINICAL OFFICER AT A SUBDISTRICT HOSPITAL

(Interviewer): You have gone through a scenario about resuscitating a child with dehydration. When you think about treating dehydration, what are the first methods that come to mind?

(CO): First you have to access the degree of dehydration then you treat according to the severity.

(Interviewer): What options do you consider when there are difficulties?

(CO): If there are difficulties you can refer.

(Interviewer): What challenges do you face when resuscitating a child with dehydration? (CO): Sometimes we don’t have the necessary equipment’slike IV fluids.

(Interviewer): What improves your ability to provide rehydration for children?

(CO): Given that there are enough supply I think we can manage and they should provide a way on which to admit this child.

(Interviewer): In the case scenario, you suggested using a nasogastric tube for rehydration therapy. What were your reasons for this?

(CO):Like now in a case where a child is refusing to take so you can put a nasogastric tube.

(Interviewer): Tell me about circumstances in which you would use a nasogastric tube for rehydration therapy.

(CO): When IV line fails that is an option.

(Interviewer): What would make it difficult to use an NG tube? What would make this process easier?

(CO): Actually there are no difficulties.

(Interviewer): Are there circumstances when you would not use a nasogastric tube for rehydration therapy?

(CO): If the child is severely dehydrated and diarrhea, although first line is when the child is vomiting which means there is no retention.

(Interviewer): Are NG tubes available in the setting in which you work?

(CO): Sometimes not always available.

(Interviewer): What training or experience have you had with using nasogastric tubes?

(CO): So far they have not offered any training.

(Interviewer): How familiar are you with the World Health Organization Guidelines for rehydration therapy for diarrheal illnesses?

(CO): Am actually because we have some posters of guidelines on how to clerk, first is how you classify dehydration then how you treat.

(Interviewer): How much training have you had regarding these guidelines?

(CO): I have just read alone there is no training.

(Interviewer): How do you think a child’s family would react if you wanted to use an NG tube for rehydrating their child?

(CO): There are several families who actually reject, that they don’t think that that is the best method.

(Interviewer): Are there cultural barriers?

(CO): Yes.

(Interviewer): After explaining to them why you are putting the NG tube do you think they are more receptive towards the idea?

(CO): Yes.

(Interviewer): So what are the biggest barriers you face in this set up while rehydrating children?

(CO): Like now the unavailability of the equipment.

(Interviewer): So according to your opinion do you think NG tubes are as good as IV? (CO): Yes.

(Interviewer): Thank you, that’s the end of the interview and the survey.

**INTERVIEW 29 NURSE AT A DISPENSARY**

(Interviewer): You have gone through a scenario about resuscitating a child with dehydration. When you think about treating dehydration, what are the first methods that come to mind?

(Nurse): We have the oral route and the IV but this one depends on the patient, if the child is dehydrated you must think of possibly the IV, if the child is severely dehydrated and vomiting it might be had to give oral.

(Interviewer): What options do you consider when there are difficulties?

(Nurse): The options depends on the child even if its severe but you have failed the line it means you start the oral therapy meanwhile when you refer to other facility they might be able to do other things like cut down.

(Interviewer): What challenges do you face when resuscitating a child with dehydration? (Nurse): They come in very late and when we try to assist and try to refer the clients are not ready, at times they are not ready to move to the next level even if you try to advice. And sometimes you tell the client to sit and give oral sips the mother things that this is just normal water even if you try to tell them this is rehydration it’s going to assist the child the clients are reluctant to give the oral sips.

(Interviewer): What improves your ability to provide rehydration for children?

(Nurse): Health education that is the paramount thing, in the mornings we try to advise our clients when the child is having diarrhea it’s important to bring the child early to the facility to avoid the child escalating to severe dehydration.

(Interviewer): In the case scenario, you did not suggest using a nasogastric tube for rehydration therapy. What were your reasons for this?

(Nurse): I didn’t think of that line because many clients think when you put a nasogastric to a child that child is severally sick and many of them tend to think that is somebody who is possibly passing away.

(Interviewer): Tell me about circumstances in which you would use a nasogastric tube for rehydration therapy.

(Nurse): The circumstances might be the child is severely dehydrated you might not be able to secure the line.

(Interviewer): What would make it difficult to use an NG tube?

(Nurse): If the mother will not be able to give in so that we fix you see that is going to be hard, and most of the time when we are putting the nasogastric tube the client is supposed to assist you to swallow.

(Interviewer): What would make this process easier?

(Nurse): If the mother complies it’s going to be easier.

(Interviewer): Are there circumstances when you would not use a nasogastric tube for rehydration therapy?

(Nurse): If the child is able to take orally.

(Interviewer): Are NG tubes available in the setting in which you work?

(Nurse): Yer, all the sizes, all the time.

(Interviewer): What training or experience have you had with using nasogastric tubes?

(Nurse): Unfortunately let me say since we came to this facility we have not been able to use the NG tube.

(Interviewer): How familiar are you with the World Health Organization Guidelines for rehydration therapy for diarrheal illnesses?

(Nurse): I have not been offered any training but if I get a child who is severely dehydrated i know a bit of the steps I can use before the child is referred.

(Interviewer): How do you think a child’s family would react if you wanted to use an NG tube for rehydrating their child?

(Nurse): Most of the times they are not willing because most of them attribute that thing to a patient who is severely ill, but with enough information most of them comply. (Interviewer): So do you think training would improve on the usage of NG tubes? (Nurse): It might improve but not very much this one depends on the health education on the side of us to the client.

(Interviewer): So according to your opinion in rehydration do you think NGs are as good as IV?

(Nurse): As I said it depends on the state of the patient but most of the time IV is better, because when you are putting the NG tube at times you might fail depending on the severity of the patient, at times you might cause harm to the nasal mucosa as you are trying to put the NG tube.

(Interviewer): So that’s the end of the survey and interview. Thank you

**INTERVIEW 30 NURSE AT A DISPENSARY**

(Interviewer): You have gone through a scenario about resuscitating a child with dehydration. When you think about treating dehydration, what are the first methods that come to mind?

(Nurse): you find the cause first and then you keep rehydrating the patient. (Interviewer): What options do you consider when there are difficulties?

(Nurse): You refer the child.

(Interviewer): What challenges do you face when resuscitating a child with dehydration? (Nurse): Maybe in case of referring we don’t have a vehicle.

(Interviewer): What improves your ability to provide rehydration for children?

(Nurse): Maybe to have enough staff.

(Interviewer): In the case scenario, you did not suggest using a nasogastric tube for rehydration therapy. What were your reasons for this?

(Nurse): It is a dispensary so we have to refer to the main hospital.

(Interviewer): Tell me about circumstances in which you would use a nasogastric tube for rehydration therapy.

(Nurse): Maybe if the child cannot retain.

(Interviewer): What would make it difficult to use an NG tube?

(Nurse): The technicality of fixing.

(Interviewer): What would make this process easier?

(Nurse): To have a doctor to assist.

(Interviewer): Are there circumstances when you would not use a nasogastric tube for rehydration therapy?

(Nurse): In this facility we don’t use.

(Interviewer): Are NG tubes available in the setting in which you work?

(Nurse): We have never used so I don’t know if they are there.

(Interviewer): What training or experience have you had with using nasogastric tubes? (Nurse): We have not been trained.

(Interviewer): How familiar are you with the World Health Organization Guidelines for rehydration therapy for diarrheal illnesses?

(Nurse): IF a child comes with diarrhea you rehydrate with ORS you don’t give IV fluids. (Interviewer): How much training have you had regarding these guidelines?

(Nurse): Not yet.

(Interviewer): How do you think a child’s family would react if you wanted to use an NG tube for rehydrating their child?

(Nurse): They will feel maybe you want to hurt the child but we have never used in the facility.

(Interviewer): What are the biggest barriers you face in this facility while rehydrating children.

(Nurse): We don’t use IV fluids maybe we don’t have admissions.

(Interviewer): So do you think NG tubes according to your opinion are as good as IV? (Nurse): We have never tried so I can’t tell.

(Interviewer): So that’s the end of the interview and survey. Thank you

INTERVIEW 31 NURSE AT A DISPENSARY

(Interviewer): You have gone through a scenario about resuscitating a child with dehydration. When you think about treating dehydration, what are the first methods that come to mind?

(Nurse): The first method is giving ORS.

(Interviewer): What options do you consider when there are difficulties?

(Nurse): I would refer.

(Interviewer): What challenges do you face when resuscitating a child with dehydration? (Nurse): Finding the vein, failing to retain.

(Interviewer): What improves your ability to provide rehydration for children?

(Nurse): Having the equipment’s.

(Interviewer): In the case scenario, you did not suggest using a nasogastric tube for rehydration therapy. What were your reasons for this?

(Nurse): I didn’t think of that.

(Interviewer): Tell me about circumstances in which you would use a nasogastric tube for rehydration therapy.

(Nurse): When the child cannot take orally and if I fail to get the line and where there are no sizable cannulas.

(Interviewer): What would make it difficult to use an NG tube?

(Nurse):I think for now we don’t have those NG tubes.

(Interviewer):What would make this process easier?

(Nurse): When we have enough equipment’s.

(Interviewer): Are there circumstances when you would not use a nasogastric tube for rehydration therapy?

(Nurse): I have not experienced using NG tubes.

(Interviewer): Because they are not available so you don’t know.

(Nurse): Yes.

(Interviewer): What training or experience have you had with using nasogastric tubes?

(Nurse): In this setup we have never used nasogastric tubes.

(Interviewer): And have they offered you any training?

(Nurse): No.

(Interviewer): How familiar are you with the World Health Organization Guidelines for rehydration therapy for diarrheal illnesses?

(Nurse): I am not familiar.

(Interviewer): How much training have you had regarding these guidelines?

(Nurse): No training.

(Interviewer): How do you think a child’s family would react if you wanted to use an NG tube for rehydrating their child?

(Nurse): of late we have not done it but according to me they have fear, they think that you can puncture the child.

(Interviewer): Do you think they are any cultural barriers regarding nasogastric tubes to the parents?

(Nurse): No.

(Interviewer): So what are the biggest barriers you face in this setting while rehydrating children?

(Nurse): The mothers of the children are impatient.

(Interviewer): So according to your opinion do you think NGs are as good as IV?

(Nurse): I think so.

(Interviewer): So that’s the end of the interview and the survey. Thank you

**INTERVIEW 32 CLINICAL OFFICER AT A DISPENSARY**

(Interviewer)You have gone through a scenario about resuscitating a child with dehydration.

When you think about treating dehydration, what are the first methods that come to mind?

(CO): Using ORS and then zinc sulphate, and we usually give vitamin A.

(Interviewer): What options do you consider when there are difficulties?

(CO): I would consider referral.

(Interviewer): What challenges do you face when resuscitating a child with dehydration?

(CO): Inadequacy of equipment’s another thing we have challenges in referring this patients

because there are no ambulance and getting a taxi is usually cumbersome

(Interviewer): What improves your ability to provide rehydration for children?

(CO): Maybe if we have a modern ORT corner which is well equipped. And then staffs should be trained on rehydration therapy.

(Interviewer): In the case scenario, you suggested using a nasogastric tube for rehydration therapy. What were your reasons?

(CO): Because if am trained in using NG I can fix it, because this child was vomiting so it means giving ORS orally maybe ha cannot retain.

(Interviewer): Tell me about circumstances in which you would use a nasogastric tube for rehydration therapy.

(CO): Where by an IV line has failed another circumstance is whereby the child is vomiting and is trained in using the NG tube I can use it.

(Interviewer): What would make it difficult to use an NG tube?

(CO): Maybe skills for those who do not have the skills of fixing it.

(Interviewer): What would make this process easier?

(CO): Adequate training.

(Interviewer): Are there circumstances when you would not use a nasogastric tube for rehydration therapy?

(CO): Yer, as I had said when you don’t have enough skills and then this child is very sick NGT won’t be appropriate.

(Interviewer): Are NG tubes available in the setting in which you work?

(CO): No.

(Interviewer): What training or experience have you had with using nasogastric tubes? (CO): Just when I was in college and immediately after college when I was doing my internship.

(Interviewer): How familiar are you with the World Health Organization Guidelines for rehydration therapy for diarrheal illnesses?

(CO): I am not familiar with the WHO guidelines but I am familiar with IMCI, I did IMCI. (Interviewer): How much training have you had regarding these guidelines?

(CO): I have trained as a participant and a facilitator of the same

(Interviewer): How do you think a child’s family would react if you wanted to use an NG tube for rehydrating their child?

(CO): They really need counseling because they think this child is very sick.

(Interviewer): So what are the biggest barriers you face in this setting while rehydrating children?

(CO): As I said earlier room and then trained staff in rehydration.

(Interviewer): So do you think there are any cultural barriers according to you on NG tubes?

(CO): No.

(Interviewer): So according to your opinion do you think NGs are as good as IV?

(CO): No I think IV is better than NG.

(Interviewer): Okay, that’s the end of the interview and the survey. thank you,

**INTERVIEW 33 AT A DISTRICT HOSPITAL**

(Interviewer): You have gone through a scenario about resuscitating a child with dehydration. When you think about treating dehydration, what are the first methods come to mind?

(Nurse): It depends with the degree of dehydration so if it is severe I will just rush to IV cause I know it will take time but if it’s not severe I will give ORS depending on the classification,

(Interviewer): What options do you consider when there are difficulties?

(Nurse): Like here we would wish to have a casualty which is provided with equipment’s, you see as much as we would like to fix the intraosseous because we don’t have the space and also the knowledge you know we need to be told.

(Interviewer): What challenges do you face when resuscitating a child with dehydration? (Nurse): First we don’t have a casualty and by the time we think of rushing the child to referral that is time wasted and this child maybe is unable to drink, so that is our biggest challenge, so if we can get a casualty which is well equipped, we can be able to stabilize before referring.

(Interviewer): What improves your ability to provide rehydration for children?

(Nurse): Learning like maybe seminars workshop and if also the management can provide us the equipment’s.

(Interviewer): In the case scenario, you did not suggest using a nasogastric tube for rehydration therapy. What were your reasons for this?

(Nurse): I assumed this child was in shock, so before you start pushing the nasogastric it won’tsucceed.

(Interviewer): Tell me about circumstances in which you would use a nasogastric tube for rehydration therapy.

(Nurse): I can only use the nasogastric tube fast to take the shock and if the child is not able to take but is stable then I can use a nasogastric tube.

(Interviewer): What would make it difficult to use an NG tube?

(Nurse): I wouldn’t use an NG tube depending on the condition of the child. (Interviewer): What would make this process easier?

(Interviewer): If we have those tubes,

ORS,and if we have the knowledge you know you can have everything and you don’t even know how much to pump in.

(Interviewer): Are there circumstances when you would not use a nasogastric tube for rehydration therapy?

(Nurse):Yes, a child in shock.

(Interviewer): Are NG tubes available in the setting in which you work?

(Nurse): I have no seen them here.

(Interviewer): What training or experience have you had with using nasogastric tubes? (Nurse): None.

(Interviewer): How familiar are you with the World Health Organization Guidelines for rehydration therapy for diarrheal illnesses?

(Nurse): I feel I am still far I need more knowledge.

(Interviewer): How much training have you had regarding these guidelines?

(Nurse): I had one in IMCI but it was long back.

(Interviewer): How do you think a child’s family would react if you wanted to use an NG tube for rehydrating their child?

(Nurse): Some will take it positively, some negatively.

(Interviewer): So what are the biggest barriers you face in this setting while rehydrating children?

(Nurse): Space to manage the children.

(Interviewer): So do you think training on procedural skills on rehydration would improve on the management.

(Nurse): Very much.

(Interviewer): So according to your opinion are NG tubes as good as IV for rehydration. (Nurse): No, I wouldn’t use an NG tube for a child who is collapsing that is risky. (Interviewer): That’s the end of the interview and the survey. Thank you

INTERVIEW 34 NURSE AT A DISTRICT HOSPITAL.

(Interviewer): You have gone through a scenario about resuscitating a child with dehydration. When you think about treating dehydration, what are the first methods that come to mind?

(Nurse):Oral rehydrating salts, encouraging breastfeeding.

(Interviewer): What options do you consider when there are difficulties?

(Nurse): Referral.

(Interviewer): What challenges do you face when resuscitating a child with dehydration? (Nurse): I can’tsay because I have encountered any.

(Interviewer): What improves your ability to provide rehydration for children?

(Nurse): Encourage fluid uptake.

(Interviewer): In the case scenario, you did not suggest using a nasogastric tube for rehydration therapy. What were your reasons for this?

(Nurse): I am not sure of my reasons, but NGT is another option.

(Interviewer): Tell me about circumstances in which you would use a nasogastric tube for rehydration therapy.

(Nurse): When the baby is not able to take fluids orally.

(Interviewer): What would make it difficult to use an NG tube?

(Nurse): There is no difficulty.

(Interviewer): Are there circumstances when you would not use a nasogastric tube for rehydration therapy?

(Nurse): Maybe the child is able to feed and not severely dehydrated.

(Interviewer): Are NG tubes available in the setting in which you work?

(Nurse):I have not seen them in this setting.

(Interviewer): What training or experience have you had with using nasogastric tubes? (Nurse): I can only say am experienced.

(Interviewer): How familiar are you with the World Health Organization Guidelines for rehydration therapy for diarrheal illnesses?

(Nurse):I am familiar but I have not mastered it that much.

(Interviewer): How much training have you had regarding these guidelines?

(Nurse): No I haven’t.

(Interviewer): How do you think a child’s family would react if you wanted to use an NG tube for rehydrating their child?

(Nurse): They are shocked they cannot take it but they are some for the idea because you want to save the kid.

(Interviewer): So according to your opinion do you think NGs are as good as IV?

(Nurse): For resuscitation IV is good.

(Interviewer): So that’s the end of the interview and the survey thank you.

INTERVIEW 35 NURSE AT A DISTRICT HOSPITAL.

(Interviewer):You have gone through a scenario about resuscitating a child with dehydration. When you think about treating dehydration, what are the first methods that come to mind?

(Nurse): Fluids, oral first.

(Interviewer): What options do you consider when there are difficulties?

(Nurse): ORS.

(Interviewer): What challenges do you face when resuscitating a child with dehydration? (Nurse): In our setup we don’t have an ORT corner so we put them outside under a tree, so sometimes it’s difficult to monitor under a tree.

(Interviewer): What improves your ability to provide rehydration for children?

(Nurse): Space.

(Interviewer): In the case scenario, you did not suggest using a nasogastric tube for rehydration therapy. What were your reasons for this?

(Nurse): I am not sure if we have any NG tubes here.

(Interviewer): Tell me about circumstances in which you would use a nasogastric tube for rehydration therapy.

(Nurse): I am not aware if anyone is supposed to use an NG tube for rehydration.

(Interviewer): Are NG tubes available in the setting in which you work?

(Nurse): No.

(Interviewer): What training or experience have you had with using nasogastric tubes?

(Nurse): The only training that I got is back in college.

(Interviewer): How familiar are you with the World Health Organization Guidelines for rehydration therapy for diarrheal illnesses?

(Nurse): I think what I know is in case of dehydration mostly we normally we go for if the kid can take anything you give ORS and then IV fluids.

(Interviewer): How much training have you had regarding these guidelines?

(Nurse): None.

(Interviewer): How do you think a child’s family would react if you wanted to use an NG tube for rehydrating their child?

(Nurse): I haven’t experienced but I don’t think most of them will like it.

(Interviewer): So in this setting what are the biggest barriers you face while rehydrating children?

(Nurse): Our biggest challenge is the space, and also the knowledge because most of us haven’t trained.

(Interviewer): Okay, thank you that’s the end of the interview and the survey.

INTERVIEW 36 NURSE AT A DISTRICT HOSPITAL

(Interviewer):You have gone through a scenario about resuscitating a child with dehydration. When you think about treating dehydration, what are the first methods that come to mind?

(Nurse): To rehydrate the quantity of water in the system.

(Interviewer): What options do you consider when there are difficulties?

(Nurse): NG tube or ORS.

(Interviewer): What challenges do you face when resuscitating a child with dehydration? (Nurse): there is no pediatrician or a doctor or room for rehydration.

(Interviewer): What improves your ability to provide rehydration for children?

(Nurse): I think updating on management.

(Interviewer): In the case scenario, you suggested using a nasogastric tube for rehydration therapy. What were your reasons for this?

(Nurse): Absorption is easier.

(Interviewer): Tell me about circumstances in which you would use a nasogastric tube for rehydration therapy.

(Nurse): Severe and when the line has failed and when the child is unable to swallow.(Interviewer): What would make it difficult to use an NG tube?

(Nurse): Unless uncooparation from the parents.

(Interviewer): What would make this process easier?

(Nurse): Counseling the parents to accept the method of rehydration.

(Interviewer): Are there circumstances when you would not use a nasogastric tube for rehydration therapy?

(Nurse): Yer, like irritable child

(Interviewer): Are NG tubes available in the setting in which you work?

(Nurse): I have not seen them.

(Interviewer): What training or experience have you had with using nasogastric tubes? (Nurse): At times they coil they don’t go to the stomach.

(Interviewer): Have you had any training regarding the usage of NG tubes?

(Nurse): No

(Interviewer): How familiar are you with the World Health Organization Guidelines for rehydration therapy for diarrheal illnesses?

(Nurse): I am familiar.

(Interviewer): How much training have you had regarding these guidelines?

(Nurse): I have not heard any training, just job experience.

(Interviewer): How do you think a child’s family would react if you wanted to use an NG tube for rehydrating their child?

(Nurse):They tend to fear that you can cause more harm to the child.

(Interviewer): So after explaining to them why you are doing it do you think they become more positive towards the idea.

(Nurse): They become positive.

(Interviewer): What are the biggest barriers you face in this setting while rehydrating children?....... like cultural barriers, maybe parents have a cultural notion against the usage of NG tubes or any other method of treating dehydration.

(Nurse):You know in the community where we are when they see you passing a nasogastric tube they say they are very sick and you are going to cause more harm.

(Interviewer): According to your opinion do you think NG tubes are as good as IV? (Nurse): Infarct NG is better than IV according to me.

(Interviewer): So that’s the end of the interview and survey. Thank you

INTERVIEW 37 NURSE AT A DISTRICT HOSPITAL.

(Interviewer): You have gone through a scenario about resuscitating a child with dehydration. When you think about treating dehydration, what are the first methods that come to mind?

(Nurse): Give ORS.

(Interviewer): What options do you consider when there are difficulties?

(Nurse): Guess where there is difficulty in drinking we give IV fluids.

(Interviewer): What challengers do you face when resuscitating a child with dehydration?

(Nurse): To get a line sometimes it’s difficult.

(Interviewer): What improves your ability to provide rehydration for children?

(Nurse): Incase the children are able to take and not vomit we give and monitor the ORS.

(Interviewer): In the case scenario, you did not suggest using a nasogastric tube for rehydration therapy. What were your reasons for this?

(Nurse): Maybe the NGT has failed and I was not able to fix the NGT.

(Interviewer): Tell me about circumstances in which you would use a nasogastric tube for rehydration therapy.

(Nurse): Maybe when there are sores in the mouth and the child cannot swallow. (Interviewer): What would make it difficult to use an NG tube?

(Nurse): Incase am not able to fix the NGT.

(Interviewer):What would make this process easier?

(Nurse): Practice.

(Interviewer): Are there circumstances when you would not use a nasogastric tube for rehydration therapy?

(Nurse): In case there is a problem with digestion.

(Interviewer): Are NG tubes available in the setting in which you work?

(Nurse): Very rare because we don’t have admission wards.

(Interviewer): According to your opinion do you think NGs are as good as IV?

(Nurse): Yer as long as the child can tolerate.

(Interviewer): So that’s the end of the interview and the survey. Thank you.

(Interviewer): What training or experience have you had with using nasogastric tubes? (Nurse): I have not had any training but I have experience of using nasogastric tubes since I was working in a health center and there we used to fix NG tubes.

(Interviewer); How familiar are you with the World Health Organization Guidelines for rehydration therapy for diarrheal illnesses?

(Nurse): Not very familiar.

(Interviewer): How much training have you had regarding these guidelines?

(Nurse): No training.

(Interviewer): How do you think a child’s family would react if you wanted to use an NG tube for rehydrating their child?

(Nurse): They will not feel nice because of the way the child will behave when you fix the NG tube.

(Interviewer): After explaining to them why you are fixing the nasogastric tube do you think they will become more positive towards the idea?

(Nurse): Yer.

(Interviewer): What are the biggest barriers you face in this setting while rehydrating children?

(Nurse): Sometimes the children cannot tolerate while using ORS and sometimes fixing a line is an issue.

(Interviewer): Do you think training of procedural skills like of fixing NG tubes will make people more prone to use NG tubes and will improve rehydration of children?

(Nurse): Yer.

(Interviewer): So according to your opinion do you think NGs are as good as IV?

(Nurse): Yer, as long as the child can tolerate and is not vomiting.

(Interviewer): So that’s the end of the interview and the survey. Thank you very much.

**INTERVIEW 39 MEDICAL OFFICER INTERN AT A REFFARAL HOSPITAL**

(Interviewer): You have gone through a scenario about resuscitating a child with dehydration. When you think about treating dehydration, what are the first methods that come to mind?

(MOI): It depends with the level but obviously I have to rehydrate either intravenous or oral.

(Interviewer): What options do you consider when there are difficulties?

(MOI): Of course accessing IV that’s the biggest challenge then secondly is the inability of the parents to take care of the line adequately, sometimes they usually careless, but I think the issue of fluids being available, they are always available all the time, so it’s just getting the IV access then maintaining the access.

(Interviewer): What improves your ability to provide rehydration for children?

(MOI): I think we need sort of a training on fixing an intraosseos line the ones we have had we have fixed but we have never had a training before so I think training many people to know that once you attempt a peripheral line and you are unable you can comfortably insert an intraosseous.

(Interviewer): In the case scenario, you suggested using a nasogastric tube for rehydration therapy. What were your reasons for that?

(MOI): You know sometimes you might delay in fixing an intraosseous and nasogastric is easy and many people can actually fix it, so in the acute phase you have to fix a nasogastric as you attempt to fix the intraosseous so it’s just to replace the fluid without wasting time.

(Interviewer): Tell me about circumstances in which you would use a nasogastric tube for rehydration therapy.

(MOI): When you are in the process of trying to get an intraosseous line so you can have a nasogastric at the same time or if someone has what seems like intestinal obstruction you can fix a nasogastric tube to decompress.

(Interviewer): What would make it difficult to use an NG tube?

(MOI): If you don’t know how to fix one, if you have the wrong size of the nasogastric tube and if the child and the care takers are unwilling. So you might fix an NG tube and you find it’s already pulled out.

(Interviewer): What would make this process easier?

(MOI): I just think education on the part of the caretakers that a nasogastric tube is actually important cause most people when they see a nasogastric tube they see someone is very sick they are about to die, so they are actually reluctant on using a nasogastric tube.

(Interviewer): Are there circumstances when you would not use a nasogastric tube for rehydration therapy?

(MOI): Yer when I easily access the peripheral line I don’t need a nasogastric tube.

(Interviewer): Are NG tubes available in the setting in which you work?

(MOI): Yer they are usually available.

(Interviewer): All the time?

(MOI): Most of the time.

(Interviewer): What training or experience have you had with using nasogastric tubes? (MOI): The training I had was in medical school and the training I had was basically in all the rotation you have to fix the NG tube.

(Interviewer): They have not offered you any sort of training?

(MOI): No.

(Interviewer): How familiar are you with the World Health Organization Guidelines for rehydration therapy for diarrheal illnesses?

(MOI): I am familiar with them.

(Interviewer): How much training have you had regarding these guidelines?

(MOI): The only training I had is in medical school.

(Interviewer): How do you think a child’s family would react if you wanted to use an NG tube for rehydrating their child?

(MOI): Most of them are reluctant as I said, people think that when you fix the NG tube the child is sick they are about to die so most of them are resistant at first.

(Interviewer): So do you think training on procedural skills would improve on the usage of nasogastric tube?

(MOI): Yer it will.

(Interviewer): So according to your opinion do you think NG tubes are as good as IV in rehydration?

(MOI): They are not as good as IV especially when treating shock.

(Interviewer): So that’s the end of the interview and the survey. Thank you very much.

**INTREVIEW 40 REGISTRAR AT A REFARAL HOSPITAL**

(Interviewer): You have gone through a scenario about resuscitating a child with dehydration. When you think about treating dehydration, what are the first methods that come to mind?

(Registrar): Oral.

(Interviewer): What options do you consider when there are difficulties?

(Registrar): The first one usually the NG tube and then intraosseous.

(Interviewer): What challenges do you face when resuscitating a child with dehydration? (Registrar): Sometimes it’s the availability of the material you want sometimes it takes too long before you are given what you want or they give you wrong items.

(Interviewer): What improves your ability to provide rehydration for children? (Registrar): If we had a standard way of doing it, like if we had a protocol, maybe have a specific area where we do it, like if we have a patient who is dehydrated I would know this is the room to take and this are the requirements, if it can be standardized I think that will also help.

(Interviewer): In the case scenario, you suggested using a nasogastric tube for rehydration therapy. What were your reasons for this?

(Registrar): Okay, it’s easy to put then the issue of volume doesn’t really worry you so much and NG tubes usually most of the time are always available and then compared to the intraosseous where you are worried of the risk if you give too much again you might cause problems to the bone marrow and absorption also I think it’s pretty good.

(Interviewer): Tell me about circumstances in which you would use a nasogastric tube for rehydration therapy.

(Registrar): Okay just like for that case scenario, maybe I don’t have the intraosseous needle or maybe the IV fluids are not available.

(Interviewer): What would make it difficult to use an NG tube?

(Registrar): Okay a baby who is still vomiting a lot if I give it most of it will be coming out and then a child who is very sick maybe like acidotic and impaired blood supply to the gut therefore poor absorption.

(Interviewer): What would make this process easier?

(Registrar): I think just the availability of the requirement like the NG tube itself and having the IV fluids available.

(Interviewer): Are there circumstances when you would not use a nasogastric tube for rehydration therapy?

(Registrar): any circumstances…………………….. I don’t think I know.

(Interviewer): Something maybe like contraindications or where you want to use a nasogastric tube but you can’t.

(Registrar): I don’t know.

(Interviewer): Are NG tubes available in the setting in which you work?

(Registrar): They are available but not always especially the current requirement where you have to order one item for each patient.

(Interviewer): What training or experience have you had with using nasogastric tubes? (Registrar): I haven’t used it much when it comes to oral rehydration but a few times we have tried it like you get a child who is severely dehydrated and you can’t get the line so I give him fluids, there was a case I actually got this child was severely dehydrated and was a chubby one and getting a line was difficult so we gave via NGT and it actually worked. (Interviewer): So have they offered you any training on the usage of NG tubes? (Registrar): Just the basic training I had no special training.

(Interviewer): How familiar are you with the World Health Organization Guidelines for rehydration therapy for diarrheal illnesses?

(Registrar): I think I am well conversant with it.

(Interviewer): How much training have you had regarding these guidelines?

(Registrar): Okay through IMCI, reading their manuals, and I also covered it as an undergraduate student.

(Interviewer): How do you think a child’s family would react if you wanted to use an NG tube for rehydrating their child?

(Registrar): Okay majority I don’t think they will mind, but occasionally a few associate tubes with bad things happening to their children and a child who is about to die but majority I think they will cooperate.

(Interviewer): Do you think they have any cultural barriers towards the nasogastric tube?

(Registrar): Probably a few.

(Interviewer): What are the biggest barriers you face in this setting while rehydrating children?

(Registrar): I think the problem here is with the staffing, like the nurses here have many children here to attend to and then the issue of having a specific protocol we all use, (Interviewer): So do you think training on procedural training would improve on rehydration?

(Registrar): Yes.

(Interviewer): So according to your opinion do you think NGs are as good as IV in rehydration?

(Registrar): Yer they are.

(Interviewer): So that’s the end if the interview and the survey. Thank you very much.

1. INTERVIEW 41 REGISTRA AT A REFARAL HOSPITAL
2. (Interviewer): You have gone through a scenario about resuscitating a child with dehydration. When you think about treating dehydration, what are the first methods that come to mind?
3. (Registrar): First method is IV then oral then intraosseous. You start with oral then IV then intraosseous.
4. (Interviewer): What options do you consider when there are difficulties?
5. (Registrar): Intraosseous.
6. (Interviewer): What challenges do you face when resuscitating a child with dehydration? (Registrar): Getting an intraosseous that cannula in pediatric is not easy so we adapt we use another we use another line gauge, so we don’t have an intraosseous.
7. (Interviewer): What improves your ability to provide rehydration for children? (Registrar): Getting that line especially in shock, then the others are beyond, me early referral, patients coming in early with dehydration.
8. (Interviewer): In the case scenario, you did not suggest using a nasogastric tube for rehydration therapy. What were your reasons for this?
9. (Registrar): Because the gut is poorly supplied so intraosseous is far much better than NG.
10. (Interviewer): Tell me about circumstances in which you would use a nasogastric tube for rehydration therapy.
11. (Registrar): Now if I don’t have any sort of thing to use, if I don’t have a line, there is no cannula available, nothing to modify to use an intraosseous and there is a tube you can put, so that’s when you can use an NG tube.
12. (Interviewer): What would make it difficult to use an NG tube?
13. (Registrar): The mother refusing.
14. (Interviewer): What would make this process easier?
15. (Registrar): Education of the parent because the mother at that time will accept because the child is sick, so fixing we can fix there actually no problem there maybe not having the nasogastric tube.
16. (Interviewer): Are there circumstances when you would not use a nasogastric tube for rehydration therapy?
17. (Registrar): Year, if you have a line, you have the IV access you don’t need the nasogastric and if you have intraosseous access you don’t need a nasogastric.
18. (Interviewer): Are NG tubes available in the setting in which you work?
19. (Registrar): Yes they are.
20. (Interviewer): All the time?
21. (Registrar): year.
22. (Interviewer): All the sizes?
23. (Registrar): Not all the sizes but they are always available.
24. (Interviewer): What training or experience have you had with using nasogastric tubes? (Registrar): There have been no training of fixing nasogastric most of us learn by watching and performing.
25. (Interviewer): So they have not offered any training?
26. (Registrar): No.
27. (Interviewer): How familiar are you with the World Health Organization Guidelines for rehydration therapy for diarrheal illnesses?
28. (Registrar): I am familiar.
29. (Interviewer): How much training have you had regarding these guidelines?
30. (Registrar): I have never undergone any training.
31. (Interviewer): How do you think a child’s family would react if you wanted to use an NG tube for rehydrating their child?
32. (Registrar): It depends on how sick the child is, if the child is very sick and the parents can see the child is sick but if he is not that sick they will not allow us to use the tube. (Interviewer): So after taking their consent and explaining to them why you are using the NG tube do you think they will be more receptive.
33. (Registrar): Year most of them are.
34. (Interviewer): What are the biggest barriers you face in this setting while rehydrating children?
35. (Registrar): Facilities………. The equipment’s.
36. (Interviewer): Do you think training on procedural skills will improve rehydration. (Registrar): Obviously it will, there is nothing wrong with training.
37. (Interviewer): So according to your opinion do you think NG tubes are as good as IV? (Registrar): In my opinion NG are not as good as IV.
38. (Interviewer): So that’s the end of the interview and the survey. Thank you very much.

**INTERVIEW 42 MEDICAL OFFICER INTERN AT A REFFARAL HOSPITAL**

(Interviewer): You have gone through a scenario about resuscitating a child with dehydration. When you think about treating dehydration, what are the first methods that come to mind?

(MOI): Normally its fluids, so there are various forms of doing it, normally we have plan C, plan B, plan A. A involves fluids oral rehydration salts and different formulas some rushed, depending if you are doing maintenance.

(Interviewer): What options do you consider when there are difficulties?

(MOI): Well first is the parent factor, they come normally when the child is severely dehydrated, they don’t come early enough when you can do things to manage the child, we are infarct in a referral hospital where we have everything, so the main challenge is the parent factor when they come in they come late when the child is severely dehydrated.

(Interviewer): What improves your ability to provide rehydration for children?

(MOI): Availability, like you know the good thing in our hospital here is that we have an IVPU we produce our own fluids, so basically is having the fluids available and trained personnel, trained enough to know when to use IV fluids when to go for ORS and when to refer when need be.

(Interviewer): In the case scenario, you did not suggest using a nasogastric tube for rehydration therapy. What were your reasons for that?

(MOI): Well what I need to improve first is the volume of the child, NGT will work first if we couldn’t find either of the IV access but it has to go into the stomach get absorbed then into the circulatory system so takes a while but it works also.

(Interviewer): Tell me about circumstances in which you would use a nasogastric tube for rehydration therapy.

(MOI): Well lack of access of IV line except in a patient who has got facial burns. Or severe burns in the limbs, trunk and the only option you have is a nasogastric tube, the need for using an NG tube is what will make it difficult to use it in the first place because the child has burns, and so whatever causes you to use a nasogastric tube is what will make it difficult.

(Interviewer): What would make this process easier?

(MOI): skill level, because putting an NG tube is not that easy, I don’t think there is a particular circumstance that will make it easier.

(Interviewer): Are there circumstances when you would not use a nasogastric tube for rehydration therapy?

(MOI): If you have an IV line sure then you will not use an NG tube, it depends if the patient has basal fracture of the skull you cannot use an NG tube because if you put an NG tube it can go to the skull.

(Interviewer): Are NG tubes available in the setting in which you work?

(MOI): Yes.

(Interviewer): All the time?

(MOI): Yes.

(Interviewer): We always have.

(Interviewer): What training or experience have you had with using nasogastric tubes? (MOI): Training was med school and the rest we learn on the job, you put one comes through the mouth you put another life goes on.

(Interviewer): How familiar are you with the World Health Organization Guidelines for rehydration therapy for diarrheal illnesses?

(MOI): If it’s about the plan A B and C if it’s that am good on that one if there is something new then I am behind.

(Interviewer): How much training have you had regarding these guidelines?

(MOI): None.

(Interviewer): How do you think a child’s family would react if you wanted to use an NG tube for rehydrating their child?

(MOI): Well they will have to accept because part of our job is convincing patients, explaining to the patients what you are doing and why and I don’t think they will be any resistance if you wanted to fix an NG tube.

(Interviewer): So what do you think are the biggest barriers you face in this setup while rehydrating children?

(MOI): The state they come in, they are always to sick so apart from that staff is trained family members are not resistance so when they come to a referral hospital they know there is something wrong so there are no many other options.

(Interviewer): So according to your opinion do you think NG tubes are as good as IV while rehydrating?

(MOI): Might be as good but not as fast.

(Interviewer): So that’s the end of the interview and survey thank you very much.

(MOI): No problem, thank you too.

**INTERVIEW 43 PAEDIATRICIAN AT A REFFARAL HOSPITAL**

(Interviewer): You have gone through a scenario about resuscitating a child with dehydration. When you think about treating dehydration, what are the first methods that come to mind?

(Pediatrician): ORS.

(Interviewer): What options do you consider when there are difficulties?

(Pediatrician): One option is NG tube, the other option is that intraosseous, in case of shock or anything another option is consulting the anesthetists for a central line.

(Interviewer): What challenges do you face when resuscitating a child with dehydration? (Pediatrician): Mostly maybe not getting the IV line, sometimes we don’t have even the appropriate cannula for children and another possibility is that we run out of things.

(Interviewer): What improves your ability to provide rehydration for children? (Pediatrician): Another challenge that we have is having qualified staff in the outpatient setup, so one of them is to have somebody who is trained in rehydration of children, the other one is to improve rehydration fluids and equipment’s and availability.

(Interviewer): In the case scenario, you did not suggest using a nasogastric tube for rehydration therapy. What were your reasons for this?

(Pediatrician): Okay, maybe I forgot but it’s something I would really do.

(Interviewer): Tell me about circumstances in which you would use a nasogastric tube for rehydration therapy.

(Pediatrician): Well if I can’t get the IV line and the child is severely dehydrated I will use a nasogastric tube. I would also use a nasogastric tube in situations where let’s say that the child is unable to drink on their own but there are no contraindications. (Interviewer): What would make it difficult to use an NG tube?

(Pediatrician): One difficulty is let’s say a child is in shock and is unconscious I would want to be careful about using a nasogastric tube or risk respiratory distress. (Interviewer): What would make this process easier?

(Pediatrician): It’s not a difficult procedure so it should be easy.

(Interviewer): Are there circumstances when you would not use a nasogastric tube for rehydration therapy?

(Pediatrician): Yes, like the way I said a child in shock or a child who has high risk of aspiration.

(Interviewer): Are NG tubes available in the setting in which you work?

(Pediatrician): Yer they are.

(Interviewer): always?

(Pediatrician): I want to assume so, I cannot say always but they should be available.

(Interviewer): What training or experience have you had with using nasogastric tubes? (Pediatrician): A lot.

(Interviewer): So they have offered you training on the use of NG tubes.

(Pediatrician): yes

(Interviewer): How familiar are you with the World Health Organization Guidelines for rehydration therapy for diarrheal illnesses?

(Pediatrician): I am familiar.

(Interviewer): How much training have you had regarding these guidelines? (Pediatrician): A lot.

(Interviewer): How do you think a child’s family would react if you wanted to use an NG tube for rehydrating their child?

(Pediatrician): I don’t think they will refuse.

(Interviewer): So what are the biggest barriers you face in this setting while rehydrating children?

(Pediatrician): Let’s say the instructions are to the mothers, sometimes the mother think that the children should be nil per oral, sometimes they want fluids some mothers don’t want to give their children ORS they want you to put a line, especially in private practice they force you to do it so that’s one, another one is running out of things if the child is vomiting.

(Interviewer): So according to your opinion do you think NG tubes are as good as IV in rehydrating?

(Pediatrician): I think they are good except where you have to give large volumes then it can become a problem.

(Interviewer): So thank you very much that is the end of the interview and the survey.

**INTERVIEW 44 MEDICAL OFFICER INTERN AT A REFFARAL HOSPITAL**

(Interviewer): You have gone through a scenario about resuscitating a child with dehydration. When you think about treating dehydration, what are the first methods that come to mind?

(MOI): It depends on the degree of the dehydration

(Interviewer): What options do you consider when there are difficulties?

(MOI): I would definitely have to pass in the NG tube.

(Interviewer): What challenges do you face when resuscitating a child with dehydration? (MOI): If the line are not there that is a very big challenge also depends with the age of the child because the smaller the child the tougher it becomes to get the line.

(Interviewer): What improves your ability to provide rehydration for children?

(MOI): Because the veins are already collapsed there is nothing much that can improve

(Interviewer): In the case scenario, you did not suggest using a nasogastric tube for rehydration therapy. What were your reasons for that?

(MOI): Because first of all even if you don’t get the line, at times you might find that the GI tract is not being well supplied with………. Is not well perfused because of the state of shock the child is in, however in some cases you can just get in as much fluid as you can.

(Interviewer): Tell me about circumstances in which you would use a nasogastric tube for rehydration therapy.

(MOI): If I have failed all my lines.

(Interviewer): What would make it difficult to use an NG tube?

(MOI): One is if this child is in severe shock I would not use a nasogastric tube

(Interviewer): What would make this process easier?

(MOI): For you to use a nasogastric tube the gut must have a form of perfusion

(Interviewer): Are there circumstances when you would not use a nasogastric tube for rehydration therapy?

(MOI): Yes.

(Interviewer): Are NG tubes available in the setting in which you work?

(MOI): Yes they are.

(Interviewer): Are they always available?

(MOI): Depending on the sizes there are some sizes which might not be there.

(Interviewer): What training or experience have you had with using nasogastric tubes? (MOI): Significant.

(Interviewer): How familiar are you with the World Health Organization Guidelines for rehydration therapy for diarrheal illnesses?

(MOI): I am very familiar with it.

(Interviewer): How much training have you had regarding these guidelines?

(MOI): No training.

(Interviewer): How do you think a child’s family would react if you wanted to use an NG tube for rehydrating their child?

(MOI): terribly, some parents don’t want NG tubes going through their children because it makes them look sicker.

(Interviewer): So what are the biggest barriers you face in this setup while rehydrating children?

(MOI): The biggest barrier is if I don’t get the lines completely.

(Interviewer): So according to your opinion do you think NG tubes are as good as IV in rehydration?

(MOI): No they are not.

(Interviewer): So that’s the end of the survey and interview, Thank you very much.
